# Estimating Vaccine Effectiveness Against Hospitalized Influenza During Pregnancy: Multicountry Protocol for a Retrospective Cohort Study

**DOI:** 10.2196/11333

**Published:** 2019-01-21

**Authors:** Allison L Naleway, Sarah Ball, Jeffrey C Kwong, Brandy E Wyant, Mark A Katz, Annette K Regan, Margaret L Russell, Nicola P Klein, Hannah Chung, Kimberley A Simmonds, Eduardo Azziz-Baumgartner, Becca S Feldman, Avram Levy, Deshayne B Fell, Steven J Drews, Shikha Garg, Paul Effler, Noam Barda, Stephanie A Irving, Patricia Shifflett, Michael L Jackson, Mark G Thompson

**Affiliations:** 1 Kaiser Permanente Northwest Center for Health Research Portland, OR United States; 2 Abt Associates, Inc Cambridge, MA United States; 3 ICES Toronto, ON Canada; 4 Chief Physician's Office Clalit Health Services Clalit Research Institute Tel Aviv Israel; 5 School of Public Health Curtin University Perth Australia; 6 Cumming School of Medicine University of Calgary Calgary, AB Canada; 7 Kaiser Permanente Vaccine Study Center Oakland, CA United States; 8 Alberta Health Edmonton, AB Canada; 9 Influenza Division Centers for Disease Control and Prevention Atlanta, GA United States; 10 PathWest Laboratory Medicine Western Australia Perth Australia; 11 School of Epidemiology and Public Health University of Ottawa Ottawa, ON Canada; 12 Department of Laboratory Medicine and Pathology University of Alberta Edmonton, AB Canada; 13 Western Australia Department of Health Perth Australia; 14 Kaiser Permanente Washington Health Research Institute Seattle, WA United States

**Keywords:** influenza, pregnancy, hospitalization, epidemiology, vaccines

## Abstract

**Background:**

Although pregnant women are believed to have elevated risks of severe influenza infection and are targeted for influenza vaccination, no study to date has examined influenza vaccine effectiveness (IVE) against laboratory-confirmed influenza-associated hospitalizations during pregnancy, primarily because this outcome poses many methodological challenges.

**Objective:**

The Pregnancy Influenza Vaccine Effectiveness Network (PREVENT) was formed in 2016 as an international collaboration with the Centers for Disease Control and Prevention; Abt Associates; and study sites in Australia, Canada, Israel, and the United States. The primary goal of this collaboration is to estimate IVE in preventing acute respiratory or febrile illness (ARFI) hospitalizations associated with laboratory-confirmed influenza virus infection during pregnancy. Secondary aims include (1) describing the incidence, clinical course, and severity of influenza-associated ARFI hospitalization during pregnancy; (2) comparing the characteristics of ARFI-hospitalized pregnant women who were tested for influenza with those who were not tested; (3) describing influenza vaccination coverage in pregnant women; and (4) comparing birth outcomes among women with laboratory-confirmed influenza-associated hospitalization versus other noninfluenza ARFI hospitalizations.

**Methods:**

For an initial assessment of IVE, sites identified a retrospective cohort of pregnant women aged from 18 to 50 years whose pregnancies overlapped with local influenza seasons from 2010 to 2016. Pregnancies were defined as those that ended in a live birth or stillbirth of at least 20 weeks gestation. The analytic sample for the primary IVE analysis was restricted to pregnant women who were hospitalized for ARFI during site-specific influenza seasons and clinically tested for influenza virus infection using real-time reverse transcription polymerase chain reaction.

**Results:**

We identified approximately 2 million women whose pregnancies overlapped with influenza seasons; 550,344 had at least one hospitalization during this time. After restricting to women who were hospitalized for ARFI and tested for influenza, the IVE analytic sample included 1005 women.

**Conclusions:**

In addition to addressing the primary question about the effectiveness of influenza vaccination, PREVENT data will address other important knowledge gaps including understanding the incidence, clinical course, and severity of influenza-related hospitalizations during pregnancy. The data infrastructure and international partnerships created for these analyses may be useful and informative for future influenza studies.

**International Registered Report Identifier (IRRID):**

DERR1-10.2196/11333

## Introduction

### Background

Pregnant women are believed to be at greater risk of severe complications from influenza infection than nonpregnant women of childbearing age, based on findings from studies primarily conducted during influenza pandemics [1,2]. Anatomic, immunologic, and physiologic changes during pregnancy that affect respiratory, cardiovascular, and other organ systems might increase the risk and severity of infections, including influenza [3,4]. The risk of hospitalization due to clinically diagnosed influenza or pneumonia appears to increase with each trimester of pregnancy [5-7]. The vulnerability of pregnant women to severe influenza disease was observed during the 2009 A (H1N1) pandemic [8-10] and at least two prior pandemics [11,12]. However, there are substantial gaps in our knowledge regarding the seasonal burden of influenza among pregnant women.

Influenza vaccination is an effective method of influenza prevention, but the vaccine is widely underutilized during pregnancy [13-15]. Although there are ample data on the safety of inactivated influenza vaccination (IIV) during pregnancy [16-18], a major challenge to maternal immunization policy making has been the paucity of data regarding the effectiveness of IIV in preventing severe influenza-related outcomes in pregnant women [2,19]. Serologic studies have found a similar antibody response to the vaccine among pregnant and nonpregnant women [20,21]. Several observational studies have compared the rates of nonspecific respiratory illness among vaccinated and unvaccinated pregnant women with mixed results [20,22-25]. Randomized controlled trials [24,26] and observational studies in pregnant women [27,28] have reported that IIV reduces the risk of mild to moderately severe laboratory-confirmed influenza illness by about half.

### Objectives

No study to date has examined influenza vaccine effectiveness (IVE) against laboratory-confirmed influenza-associated hospitalization during pregnancy. This is an important gap in knowledge for maternal immunization policy. Perhaps the greatest challenge in addressing this IVE question is identifying study populations with sufficient numbers of influenza-related hospitalizations during pregnancy in nonpandemic seasons. Randomized controlled trials and prospective observational studies are impractical due to the large number of women that would be required to observe a statistically meaningful number of hospitalizations. In addition, randomized controlled trials may be unethical in high-income countries where influenza vaccination is recommended for pregnant women. Large-scale retrospective observational studies may be feasible, but no single public or private health care database with influenza testing results and influenza vaccination records is large enough to adequately address the question. After considering these limitations, the US Centers for Disease Control and Prevention (CDC) reached out to international partners to build a collaboration capable of determining IVE against hospitalization in pregnant women. In addition to addressing the primary IVE question, the collaboration was envisioned as a way to explore other important gaps in our understanding of influenza infection and vaccination during pregnancy.

## Methods

### Overview

The Pregnancy Influenza Vaccine Effectiveness Network (PREVENT) was formed in 2016 as an international collaboration with the US CDC, Abt Associates, and study sites in 4 countries: Australia, Canada, Israel, and the United States. PREVENT was established to address multiple gaps in knowledge about influenza vaccination and infection during pregnancy (Textbox 1). The primary goal of this collaboration was to estimate IVE in preventing acute respiratory or febrile illness (ARFI) hospitalizations associated with laboratory- confirmed influenza virus infection during pregnancy.

**Study goals and features intended to address specific knowledge gaps.**

Primary analysis: assess inactivated influenza vaccine effectiveness (IVE) in preventing severe influenza disease during pregnancy

Knowledge gap:

- No study to date has examined IVE against laboratory-confirmed influenza hospitalization during pregnancy
- Information is limited on how IVE during pregnancy may vary across seasons and by influenza type and subtype
- Information is needed on whether influenza vaccinations received in previous seasons (before pregnancy) affect IVE during pregnancy

Study feature:

- Assess IVE against laboratory-confirmed influenza-associated hospitalization using the test-negative design
- Assess IVE by site and study season and across seasons by influenza type and subtype
- Where prior season vaccination records are available, assess IVE by combinations of current and prior season influenza vaccination status

Secondary analysis: assess the frequency of hospitalization for acute respiratory and febrile illness (ARFI) associated with laboratory-confirmed influenza virus infection during pregnancy

Knowledge gap:

- Studies of influenza during pregnancy using laboratory-confirmed outcomes are scarce
- Studies of influenza-associated hospitalization during pregnancy have been predominantly limited to the United States
- Studies often enroll only during peak periods of virus circulation
- Information is limited on atypical and nonrespiratory disease manifestations of influenza virus infection
- Information on the burden of influenza disease associated with seasonal influenza viruses during pregnancy is limited, especially for severe disease requiring hospitalization

Study feature:

- Identify hospitalizations during influenza season among pregnant women with clinical testing for influenza by real-time reverse transcriptase polymerase chain reaction assay (rRT-PCR)
- Examine influenza-associated hospitalizations in regions of Australia, Canada, Israel, and the United States
- Examine influenza-associated hospitalizations during early, peak, and late periods of influenza circulation
- Assess the frequency of influenza virus infections among women hospitalized without influenza or pneumonia diagnoses, including febrile-only and sepsis-like syndromes
- Assess the incidence of influenza-associated hospitalization during pregnancy over multiple influenza seasons by study site

Secondary analysis: describe the clinical features of influenza-associated hospitalization during pregnancy

Knowledge gap:

- Frequency and application of clinical influenza testing among pregnant women hospitalized with acute respiratory illness during influenza season is unknown
- Information on the clinical epidemiology of severe influenza disease during pregnancy is scarce, especially for seasonal influenza
- Further research is needed to identify risk factors for very severe influenza disease during pregnancy that requires intensive care
- Information on the clinical course of influenza virus infections among pregnant women during hospitalization is limited, especially for those with laboratory-confirmed seasonal influenza
- Variation in illness severity and outcomes among influenza virus type and subtype has not been assessed among pregnant women with seasonal influenza
- Information on the frequency of deliveries among women hospitalized with influenza is limited

Study feature:

- Assess the frequency of clinical influenza testing across health care systems and countries and compare characteristics of tested versus untested pregnant women and their reasons for hospitalization
- Describe the characteristics of pregnant women (eg, age, trimester, underlying health conditions) hospitalized with influenza virus infection and their clinical diagnoses
- Assess the characteristics of pregnant women with influenza virus infection who are admitted to an intensive care unit (ICU) during hospitalization
- Describe the length of stay in the general ward or ICU and the frequencies of pneumonia diagnosis, respiratory failure, and need for intensive care of pregnant women hospitalized with influenza
- Compare indicators of illness severity and selected hospitalization outcomes among women with laboratory-confirmed influenza by type and subtype
- Assess the frequency of deliveries during hospitalizations with ARFI diagnoses associated with maternal influenza virus infection

Secondary analysis: describe the frequency and clinical features of respiratory syncytial virus (RSV)-associated hospitalization during pregnancy

Knowledge gap:

- Despite substantial evidence highlighting the burden of RSV in young children, little is known about RSV infection during pregnancy
- Few studies have documented the impact of antenatal RSV infection on birth outcomes

Study feature:

- Describe the clinical characteristics of RSV infection during pregnancy
- Describe outcomes at birth for women testing positive for RSV during pregnancy compared with women who test negative

Secondary analysis: examine birth outcomes associated with hospitalized influenza infection during pregnancy

Knowledge gap:

- Perinatal risks posed by antenatal influenza virus infection are unclear, especially for seasonal influenza
- Few comparative studies have accounted for gestational timing of influenza infection when comparing birth outcomes in influenza-infected and uninfected women

Study feature:

- Compare birth outcomes of women hospitalized with ARFI with laboratory-confirmed influenza virus infection with birth outcomes of women with ARFI hospitalizations confirmed as influenza-negative and women without ARFI hospitalization during pregnancy
- Compare birth outcomes by gestational age at influenza infection in women hospitalized with laboratory-confirmed influenza

Secondary analysis: assess the frequency of vaccination with inactivated influenza vaccine (IIV) during pregnancy across countries and health care systems

Knowledge gap:

- Information is limited on the uptake of IIV during pregnancy across health care systems and countries
- Information is limited on the timing of IIV vaccination, even though this has implications for the protection of the mother and the transfer of protective antibodies to the fetus
- More information is needed on the differences between IIV vaccinated versus unvaccinated pregnant women who are at greatest risk for influenza hospitalization

Study feature:

- Describe IIV coverage among women pregnant during influenza vaccine campaigns and/or influenza seasons across multiple years and study sites
- Describe the frequency of IIV vaccination among pregnant women by stage of pregnancy and relative to influenza season
- Compare the socio-demographic and underlying health characteristics of pregnant women hospitalized for ARFI during influenza season by seasonal vaccination status

Secondary aims include estimating the incidence of influenza-associated ARFI hospitalization during pregnancy, comparing the characteristics of ARFI-hospitalized pregnant women who were tested for influenza with those who were not tested, describing the clinical course and severity of influenza and noninfluenza ARFI hospitalizations during pregnancy, and describing the clinical course of respiratory syncytial virus (RSV)–associated hospitalizations during pregnancy (Textbox 1). Participating sites will also describe influenza vaccination rates in pregnant women and compare birth outcomes (such as low birth weight, preterm delivery, and small-for-gestational age births) among women with laboratory-confirmed influenza-associated hospitalizations versus other noninfluenza ARFI hospitalizations.

### Study Sites and Enrollment

In 2015, study investigators conducted a series of telephone interviews and written surveys to recruit potential international study sites. To be considered a potential PREVENT site, institutions were required to meet several inclusion criteria related to the underlying characteristics of the source population, clinical and laboratory practices, and availability of high-quality regional respiratory virus surveillance and electronic medical record (EMR) data (Textbox 2).

**Eligibility criteria for The Pregnancy Influenza Vaccine Effectiveness Network study site selection.**

- Influenza surveillance data to identify weeks of local influenza circulation for multiple years (ideally dating from 2010) were available
- Women who were pregnant during hospitalization for acute respiratory or febrile illness could be identified with electronic medical records, administrative data, or laboratory records
- Diagnostic hospital admission and/or discharge codes (International Classification of Diseases ninth revision or International Classification of Diseases, tenth revision with Australian and Canadian variations) from the records described above were accessible
- Demographic characteristics, underlying medical conditions before pregnancy, pregnancy history, and medical complications during pregnancy were available from medical records or routine registry data
- Pregnant women with acute respiratory or febrile disease during influenza season were routinely tested for influenza with real-time reverse transcriptase polymerase chain reaction (rRT-PCR) at study facilities
- Demographic characteristics, underlying medical conditions, influenza vaccination status, and clinical diagnoses of pregnant women who received clinical virus testing could be compared with those of pregnant women who were not tested during influenza season
- Influenza vaccine coverage among pregnant women in the catchment area was modest to high (10%-70%) but not universal during the study period
- Influenza vaccination records from electronic registries, electronic medical records, or public health records were available

**Table 1:** Pregnancy Influenza Vaccine Effectiveness Network study countries, sponsors, populations, and data sources.

Country (region)	Sponsoring institution	Local population (million)	Influenza seasons contributed	Pregnant women hospitalized for ARFI^a^
Australia (Western)	Western Australia Department of Health	Approximately 2.6	2012-2015 (southern hemisphere)	1639
Canada (Alberta)	Alberta Health	Approximately 4.1	2011-2015	5042
Canada (Ontario)	ICES	Approximately 14	2010-2016	7738
Israel	Clalit Health Services	Approximately 4.4	2010-2011, 2012-2016	1424^b^
United States (California, Oregon, and Washington)	Kaiser Permanente	Approximately 6.2	2011-2016	2709

^a^ARFI: acute respiratory or febrile illness.

^b^Hospitalization of pregnant women associated with deliveries that occurred in non-Clalit hospitals were not captured.

On the basis of these criteria, 5 study sites were recruited in 4 countries: Australia, Canada, Israel, and the United States (Table 1). Each PREVENT study site developed methods to define local influenza seasons, identify pregnant women, identify relevant ARFI hospitalizations and influenza tests, and extract data about influenza vaccinations and important covariates. Influenza vaccination is recommended and available at no cost to women in all study sites. A brief description of each study site and the methods they used are provided below.

#### Australia

Western Australia (WA) is the country’s largest state in total land area and has about 2.6 million residents, most of whom live in the capital city of Perth. The Western Australia Department of Health has access to data on the annual birth cohort of approximately 34,000 through its state perinatal data collection, the Midwives Notification System. This data collection includes information on >99% of births in the state with gestation ≥20 weeks (live and stillborn) and was used to identify a cohort of pregnant women who gave birth between January 2012 and December 2015. Inpatient records for all public and private hospitals in WA are available in the Hospital Morbidity Data System, which collects information on hospital discharge and was used to identify ARFI hospitalizations. Public immunization providers report influenza vaccines administered to pregnant women to the WA Antenatal Influenza Vaccination Database; the number of pregnant women obtaining vaccines in the private market is thought to be about 4% [29]. An evaluation of this dataset showed it captured 46% of the self-reported influenza vaccinations among a sample of postpartum women surveyed in WA [30]. Influenza surveillance data were obtained from 2 sources: (1) the state’s public health reference laboratory (PathWest Laboratory Medicine WA) and (2) state notifications of laboratory-confirmed influenza infection (WA Notifiable Infectious Disease Database). Laboratory real-time reverse transcription polymerase chain reaction (rRT-PCR) testing data from the state’s public health reference laboratory were linked to the cohort to identify influenza and RSV testing results.

#### Canada

Overall, 2 provinces in Canada are participating in PREVENT. The province of Alberta has about 4.1 million residents and 53,500 annual births. The Alberta Ministry of Health (Alberta Health) administers its publicly funded health care system. Each resident registered in the insurance plan has a unique lifetime identifier that can be used to link the data sources described below, including the provincial vaccination repository and the vital statistics registry. All live and stillbirths of at least 20 weeks’ gestation are available through the provincial Vital Statistic Registry, which was used to identify pregnancies. The Canadian Institute for Health Information’s Discharge Abstract Database (DAD) captures administrative, clinical, and demographic information on hospital discharges directly from all 106 acute care facilities in the province and was used to identify ARFI hospitalizations. All Albertans are eligible to receive influenza vaccination free of charge, with less than 10% of influenza vaccinations not reported to the registry. Influenza surveillance is conducted continuously in Alberta with year-round laboratory testing and a community-based sentinel physician network, hospital, and emergency room surveillance. Information about clinician-ordered influenza testing with rRT-PCR was obtained through the centralized Provincial Laboratory Information System.

The province of Ontario has about 14 million residents and approximately 147,000 births annually and includes Canada’s capital (Ottawa) and Canada’s largest city (Toronto). The sponsoring organization, The ICES, is a not-for-profit research institute whose mandate is to enable health system evaluation and research within Ontario. Data from the DAD were extracted to identify pregnant women (using their delivery hospitalization abstract) and ARFI hospitalizations. Physician and pharmacist (starting in 2012) billing claims contained in the Ontario Health Insurance Plan (OHIP) and Ontario Drug Benefits databases, respectively, were used to identify influenza vaccinations. A previous validation study of one of these sources (OHIP) found physician billing claims were 42% sensitive among pregnant women compared with self-reported influenza vaccination status, as individuals can also receive influenza vaccination through public health and workplace clinics [31]. Respiratory specimen results from Public Health Ontario and 8 academic hospital laboratories using rRT-PCR were individually linked to the health administrative data using unique encoded identifiers.

#### Israel

Clalit Health Services is the largest health care fund in Israel, covering 53% of Israel’s population. About 4.4 million people are covered by the fund, including about 93,000 births annually. Nearly all patients (>98%) remain in the fund from year to year, receiving all of their publicly funded health care through the fund. Clalit’s comprehensive EMR has been universally adopted among all inpatient and outpatient health care facilities. All live births are captured through hospital EMR data and a demographic registry that feeds into the Clalit data warehouse. An algorithm based on diagnostic and procedure codes was employed to identify pregnancies ≥20 weeks’ gestation that did not end in live births. Hospitalizations of pregnant women associated with deliveries that occurred in non-Clalit hospitals (over half to two-thirds of all hospitalizations) were not captured by the EMR. Influenza vaccines are offered free of charge to health care fund members at Clalit clinics, and details regarding influenza vaccination are entered into the EMR. Influenza and RSV testing is conducted using rRT-PCR in Clalit hospitals at the discretion of the physician, and rRT-PCR results are captured in the EMR.

#### United States

Kaiser Permanente (KP) is an integrated health care delivery system serving over 12 million people in the United States. Moreover, 3 KP sites contributed data to PREVENT—KP Northwest (Portland, OR), KP Northern California (Sacramento, San Francisco Bay Area, Fresno), and KP Washington (Seattle, WA; formerly Group Health Cooperative). The combined population of the KP PREVENT sites is about 6.2 million people, including about 56,000 live births annually. KP Northern California provides inpatient care at 21 KP-owned hospitals, KP Northwest provides inpatient care at 2 KP-owned hospitals and contracts with several other regional hospitals, and KP Washington does not own any hospitals but contracts with regional hospitals for patient care. A common comprehensive EMR system is used at the KP sites. The KP sites identified pregnancies of at least 20 weeks’ gestation using a combination of local pregnancy registry data and a validated algorithm that uses diagnosis and procedure codes to identify pregnancy episodes [32]. In addition, 2 sites, KP Washington and KP Northwest, further manually reviewed the medical records of women who were hospitalized with ARFI and excluded those not found to be pregnant. Influenza vaccination records were extracted from the EMR and from state immunization registries in Oregon and Washington states. A previous study found that KP EMR records were 89% sensitive among pregnant women compared with self-reported influenza vaccination status [27]. Influenza surveillance data for Region 10 of the United States were provided by CDC and were used to identify influenza seasons for the KP sites [33]. At KP Northern California and KP Northwest, clinical influenza and RSV rRT-PCR testing dates and results were extracted directly from the EMR. At KP Washington, test dates and results were manually abstracted from medical records.

### Influenza Seasons and Peak Period Definitions

With study sites located around the globe and in both hemispheres, a necessary first step in developing the study protocol was to agree upon a shared method for defining influenza seasons and peak periods of circulation. Each site included up to 6 seasons of data starting with the northern hemisphere 2010-2011 season as the earliest. Using a combination of regional surveillance and clinical laboratory records, each site identified the number of respiratory specimens tested and the number of laboratory-confirmed influenza positives identified among tested specimens.

Similar to previous efforts to define influenza seasons consistently across countries [34,35], each study site identified criteria to delineate the start and end of sustained influenza circulation and to identify a period of peak influenza circulation (Table 2). A total of 3 sites (Australia, Ontario [Canada], and the United States) used the mean percentage of specimens that tested positive for influenza A or B virus infection across weeks for each surveillance year to define their threshold of increased or decreased activity. Moreover, 2 sites (Alberta [Canada] and Israel) used a weekly influenza positivity rate of greater than 5% of specimens tested as their threshold.

**Table 2:** Weeks of local early, peak, and late influenza seasons; earliest and latest week of clinical influenza positives; and predominant local circulating influenza strains by year and study sites.

Region and influenza season		Range of weeks (total weeks)			Weeks, sum	Predominant local strains^a^
		Early season	Peak season	Late season		
**Northern Hemisphere 2010-2011**						
	Canada (Alberta)	50-3 (6)	4-8 (5)	9-15 (7)	18	A (H3N2)
	Canada (Ontario)	48-49 (2)	50-6 (9)	7-15 (9)	20	A (H3N2)
	Israel	48-50 (3)	51-5 (7)	6-14 (9)	19	A (H1N1)pdm; A (H3N2); B viruses
	United States (West)	51-3 (5)	4-11 (8)	12-15 (4)	17	A (H3N2); A (H1N1)pdm; B viruses
**Northern Hemisphere 2011-2012**						
	Canada (Alberta)	2-7 (6)	8-17 (10)	18-26 (9)	25	A (H3N2)
	Canada (Ontario)	5-7 (3)	8-15 (8)	16-21 (6)	17	B viruses
	United States (West)	6-8 (3)	9-20 (12)	21-25 (5)	20	A (H3N2)
**Southern Hemisphere 2012**						
	Australia (West)	27-30 (4)	31-37 (7)	38-40 (3)	14	A (H3N2)
**Northern Hemisphere 2012-2013**						
	Canada (Alberta)	46-49 (4)	50-10 (13)	11-23 (13)	30	A (H3N2), B (Yamagata)
	Canada (Ontario)	46-48 (3)	49-4 (8)	5-12 (8)	19	A (H3N2); A (H1N1)pdm
	Israel	2-3 (2)	4-8 (5)	9-14 (6)	13	A (H1N1)pdm; A (H3N2)
	United States (West)	48-51 (4)	52-10 (12)	11-20 (10)	26	A (H3N2)
**Southern Hemisphere 2013**						
	Australia (West)	33-35 (3)	36-45 (10)	46-47 (2)	15	A (H3N2); A (H1N1)pdm
**Northern Hemisphere 2013-2014**						
	Canada (Alberta)	48-51 (4)	52-6 (7)	7-10 (4)	21	A (H1N1)pdm
	Canada (Ontario)	49-49 (1)	50-11 (14)	12-22 (11)	26	A (H1N1)pdm
	Israel	52-4 (5)	5-11 (7)	12-18 (7)	19	A (H1N1)pdm; B (Yamagata)
	United States (West)	50-50 (1)	51-9 (11)	10-10 (1)	13	A (H1N1)pdm; A (H3N2)
**Southern Hemisphere 2014**						
	Australia (West)	29-31 (3)	32-40 (9)	41-44 (4)	16	A (H1N1)pdm; A (H3N2)
**Northern Hemisphere 2014-2015**						
	Canada (Alberta)	41-48 (8)	49-7 (12)	8-17 (10)	30	A (H3N2); B (Yamagata)
	Canada (Ontario)	49- 49 (1)	50-5 (9)	6-19 (14)	24	A (H3N2)
	Israel	45-3 (11)	4-9 (6)	10-10 (1)	18	A (H3N2)
	United States (West)	45-48 (4)	49-5 (10)	6-6 (1)	15	A (H3N2)
**Southern Hemisphere 2015**						
	Australia (West)	25-28 (4)	29-40 (12)	41-45 (5)	21	A (H3N2); B (Yamagata)
**Northern Hemisphere 2015-2016**						
	Canada (Ontario)	3-5 (3)	6-13 (8)	14-20 (7)	18	A (H1N1)pdm; B viruses
	Israel	49-51 (3)	52-5 (6)	6-14 (9)	18	A (H1N1)pdm; B (Victoria)
	United States (West)	52-4 (5)	5-13 (9)	14-21 (8)	22	A (H1N1)pdm; B (Yamagata)

^a^Conclusions regarding prominent strains (believed to represent >20% of circulating viruses) came primarily from real-time reverse transcriptase polymerase chain reaction assay (rRT-PCR) (sub)type results from clinical isolates from this study for Australia and Canada (Alberta and Ontario); for the United States (West) where A subtype results were not available from clinical rRT-PCR results, we referenced US Centers for Disease Control and Prevention West Coast Regional reports [33]; for Israel, where A (H1N1) pandemic (pdm) virus subtyping is consistently done but A (H3N2) virus subtyping is not, we supplemented our data with a review of clinical real-time reverse transcriptase-polymerase chain reaction results with State of Israel Ministry of Health reports [36].

With this information, each site determined for each study season:

- the *start of each season*, defined as the Sunday of the first of 3 consecutive weeks in which the percentage of specimens testing positive for influenza A or B virus infection was higher than the determined threshold;
- the *end of each season*, defined as the Saturday of the first of 3 consecutive weeks in which the percentage of specimens testing positive for influenza was below the threshold;
- the *peak period*, defined as the weeks that included ≥68% of influenza positives between the start and end of each season;
- the *early season*, defined as the weeks from the start of the season through the week before the peak period; and
- the *late season*, defined as the week after the peak period through the end of the season.

### Retrospective Cohort Identification

Pregnancies were defined as those that ended in a live birth or stillbirth of at least 20 weeks’ gestation. Sites began analysis by identifying all pregnancies during the study years (eg, starting in July of the first year and ending in June of the last year for northern hemisphere sites), with the exception of California and Washington, United States sites that could only examine pregnancies during influenza seasons. Nonetheless, among sites that attempted to identify all pregnancies during study years, 83% (1.72 million/2.07 million) of the pregnancies overlapped with an influenza season.

Study sites subsequently limited their study population to pregnant women who were hospitalized for ARFI during the site-specific influenza seasons. ARFI hospitalizations were identified using a shared list of *International Classification of Diseases, ninth and tenth revision*, diagnosis codes applied in previous studies of medically attended influenza illness [27,37,38] and expanded to include acute illnesses with febrile only, nonrespiratory, or sepsis-like presentations that may be associated with severe influenza disease among adults [39,40]. Canada and Australia used country-specific versions of these codes.

To define the analytic sample for the primary IVE analysis, we further limited the population to women who were clinically tested for influenza virus infection using rRT-PCR with respiratory specimens collected within 3 days before admission through hospital discharge. Women who were ineligible for influenza vaccination (eg, were not covered by health insurance during the vaccination campaign period), those vaccinated within 14 days of admission, and (at some sites) those without documented influenza vaccination status were excluded. ARFI hospitalizations that were readmissions within 14 days of discharge were combined with the index hospitalization and considered single events in the IVE analytic sample.

### Data Collection

As an important early step, PREVENT investigators developed and refined a shared data dictionary (Multimedia Appendix 1). Each site developed a site-specific plan to measure the common data requirements for the project. These plans were then compared and harmonized into a common set of requirements and strategies. The shared data dictionary initially focused on variables that were key to the primary aim of estimating IVE. All participating sites were able to provide all the variables in this core dataset. Additional data elements were added to support the secondary aims of the study, and for some of these secondary variables, only a subset of sites was able to provide data.

For women in the retrospective cohort, we extracted the following data from records associated with the index AFRI hospitalization and, where possible, from administrative records or ambulatory care records before hospitalization: (1) basic descriptive demographic information and maternal characteristics (eg, age, race, ethnicity, socioeconomic status, height, weight, and smoking); (2) underlying health conditions before pregnancy (eg, asthma, diabetes, and cancer); (3) pregnancy history and complications with the current pregnancy (eg, gestational hypertension and gestational diabetes; (4) clinical signs and symptoms, course, and treatment during the ARFI hospitalization; (5) respiratory specimen collection and laboratory test results for influenza, RSV, and other pathogens; (6) disposition at hospital discharge (eg, home, hospital transfer, and death); (7) delivery date, gestational age of the infant at delivery, and birth outcomes (eg, birth weight and small-for- gestational age); and (9) influenza vaccination records for the current season. When hospital or birth outcome information was not available in EMR or administrative databases, a limited medical record abstraction was performed by study sites that had direct access to medical records. During the study seasons, most sites only used trivalent inactivated influenza vaccine; quadrivalent inactivated influenza vaccine represented <5% of doses administered to pregnant women starting in 2012 in the United States and 2015 in Israel. Live, attenuated influenza vaccination is contraindicated during pregnancy and was excluded.

### Ethical Approval and Considerations

The study protocol and procedures have been reviewed and approved by institutional review boards by Abt Associates (the coordinating institution on which US CDC relies) and at each study site: Human Research Ethics Committee, Department of Health Western Australia; Conjoint Health Research Ethics Board, University of Calgary; University of Alberta Health Research Ethics Board; Sunnybrook Health Sciences Centre, Toronto, Canada; Kaiser Permanente Northwest Institutional Review Board; Clalit Health Services Research Ethics Committee. It was not possible to use a common institutional review board for this project because the institutional and regional human subjects protection policies and regulations varied for each PREVENT site.

Each site received a waiver of informed consent for all participants. The study presented minimal risk to participants, as there was no interaction or intervention with patients. Although patient information was extracted from existing administrative databases, no personal identifiers were shared between study sites Abt Associates or US CDC. Sites provided aggregate data tables that included summary statistics rather than individual-level datasets, and measures were taken to ensure subject privacy in reports with small cell sizes. There was no risk to the participants’ health from participation in this study because data were collected either as part of patients’ routine care or for billing purposes. The study had no impact on patients’ current health care or therapeutic management plan. Consequently, patients were not provided information about their participation.

## Results

**Figure 1:**
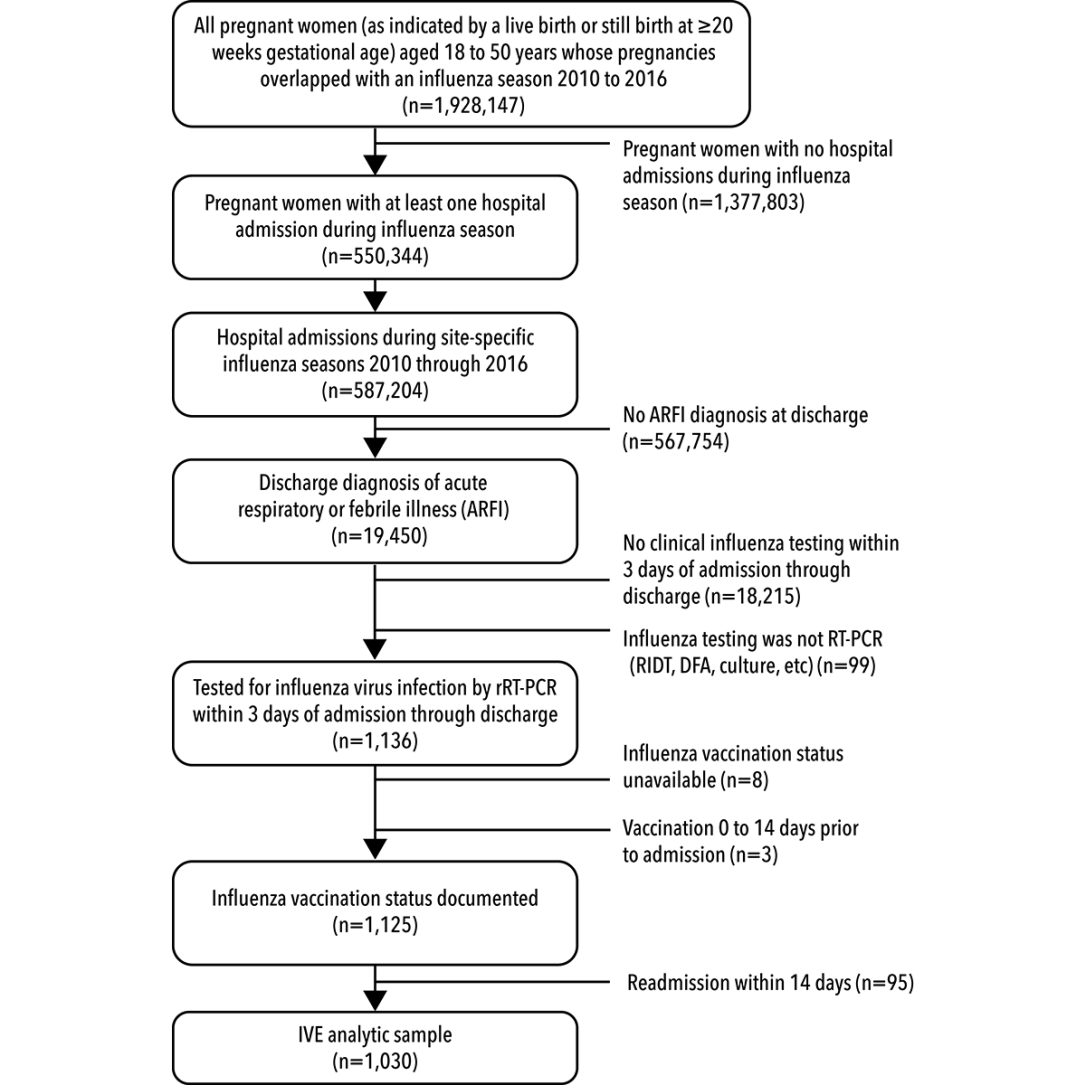
Pregnancy Influenza Vaccine Effectiveness Network retrospective inclusion and exclusion criteria. rRT-PCR: real-time reverse transcriptase polymerase chain reaction; RIDT: rapid influenza diagnostic test; DFA: direct fluorescent antibody; IVE: inactivated influenza vaccine.

Figure 1 summarizes the steps sites followed to create the retrospective PREVENT cohort, starting with pregnant women aged from 18 to 50 years at the time of inpatient admission whose pregnancies overlapped with the site-specific influenza seasons. This population of 1,928,147 pregnant women will be used in secondary analyses describing vaccination coverage, influenza incidence, and birth outcomes. To refine the sample for the IVE analysis, we limited this population to pregnant women who were hospitalized during influenza season (n=550,344). We identified 19,450 hospitalizations with ARFI diagnoses at discharge and 1136 of these included rRT-PCR influenza testing within the 3 days before admission through discharge. After excluding hospitalizations with incomplete vaccination histories and readmissions, the final IVE analytic dataset included 1030 hospitalizations and 1005 unique women.

## Discussion

### Principal Findings

The PREVENT collaboration will provide important information about the effectiveness of influenza vaccination in preventing severe laboratory-confirmed influenza illness requiring hospitalization in pregnant women and will address additional pertinent knowledge gaps about influenza and pregnancy. As hospitalization for ARFI with laboratory-confirmed influenza is a rare occurrence in pregnant women, an international collaboration was needed to address this question. Out of approximately 2 million women who were pregnant during influenza seasons 2010-2011 through 2015-2016 at 7 study sites in 4 countries, we identified about 1000 who were hospitalized and tested for influenza by rRT-PCR for inclusion in the primary IVE analysis. This analysis to address this important gap in knowledge would not be possible without an international collaboration of this magnitude.

### Strengths and Limitations

In addition to the magnitude and geographic diversity of the study cohort, this network has established resources valuable for antenatal influenza research. As part of this collaboration, PREVENT has brought together a pool of international expertise in influenza vaccination and infection during pregnancy. The study investigators worked together to develop methods to harmonize data collection, management, and analyses across different institutions and countries with differing underlying populations, data sources, and human subjects protection regulations. In addition to addressing the primary question about the effectiveness of influenza vaccination, PREVENT data will be used to address other important knowledge gaps including understanding the incidence, clinical course, and severity of hospitalized influenza during pregnancy. The data infrastructure and partnerships created for these analyses may be useful and informative for future studies.

Despite the strengths of this collaboration, there are a few limitations to the analyses within this cohort. Due to the nature of the data sources across sites, we were only able to include pregnancies ending in live birth or stillbirth of at least 20 weeks’ gestational age, because several sites were unable to extract reliable data on pregnancies ending in fetal loss before 20 weeks. We are, therefore, not able to examine the impact of influenza infection or influenza vaccination on outcomes early in pregnancy, such as spontaneous abortion. In addition, we included study sites that routinely tested pregnant women for influenza and had maternal influenza immunization programs, which limited our study to the inclusion of 4 high-income countries. Therefore, PREVENT study results may not be generalizable to countries with fewer resources dedicated to testing and vaccination programs or with different underlying population characteristics (eg, high prevalence of HIV or malaria) that may impact IVE or influenza incidence and severity. Finally, there is the potential for misclassification bias in some of our measurements. To ensure consistency across sites, chronic diseases are characterized solely by International Classification of Diseases code, which may underestimate the prevalence of these conditions in the women studied. Misclassification of influenza vaccination is of most concern for the primary IVE analysis; however, the participating sites generally have high rates of influenza vaccination capture, often using a combination of EMR data and regional and national immunization registries.

### Conclusions

Due to methodological challenges in researching seasonal influenza infection and vaccination in pregnant women, we have several important unanswered questions, including understanding the effectiveness of influenza vaccination in preventing hospitalization during pregnancy. PREVENT will address this primary IVE question as well as a number of other important gaps in our understanding of influenza and other respiratory infections during pregnancy. This work will be informative for strengthening global influenza prevention strategies and for improving the health of pregnant women.

## Appendix

Multimedia Appendix 1 Data dictionary Pregnancy Influenza Vaccine Effectiveness Network protocol.

## Abbreviations

ARFI: acute respiratory or febrile illness
CDC: Centers for Disease Control and Prevention
DAD: Discharge Abstract Database
EMR: electronic medical record
IIV: inactivated influenza vaccine
IVE: influenza vaccine effectiveness
KP: Kaiser Permanente
NH: northern hemisphere
OHIP: Ontario Health Insurance Plan
PREVENT: Pregnancy Influenza Vaccine Effectiveness Network
rRT-PCR: real-time reverse transcriptase polymerase chain reaction assay
RSV: respiratory syncytial virus
SH: southern hemisphere
WA: Western Australia
