# Supplementary material for: Estimating Vaccine Effectiveness Against Hospitalized Influenza During Pregnancy: Multicountry Protocol for a Retrospective Cohort Study
Source: JMIR Res Protoc. 2019 Jan 21;8(1):e11333. doi: 10.2196/11333 (PMC6360380; doi:10.2196/11333)
Supplement: Multimedia Appendix 1 [file resprot_v8i1e11333_app1.pdf]

INDEX

I. Specification of records included in dataset
II. Setting and Season Information
III. Acute Respiratory or Febrile Illness (ARI/F) Summary Variables from Discharge Codes (DC)
IV. Acute Respiratory or Febrile Illness (ARI/F) Specific Diagnosis Codes
V. Maternal Characteristics
VI. High Risk Medical Conditions in Discharge Codes
VII. Pregnancy History and at Admission
VIII. Hospital Event Description
IX. Illness Onset
X. Virus Testing
XI. Influenza Vaccination
XII. Birth Outcomes
XIII. Vaccination Patterns
XIV. Analysis Specification

I. Specification of Records Included in Dataset

One record per hospitalization. Each record meets all of the following criteria[i]:

a. Female age 18-50 at time of inpatient admission. Exclude any women that were less than 18 years or over 50 years at any time during the pre-defined flu season.
b. Pregnant at time of admission.
c. Date of admission during flu season 2010/11 thru 2015/16
d. Eventual pregnancy outcome of live birth or stillbirth at >= 20 weeks gestational age.
e. Discharge diagnosis of ARI/Febrile illness OR any influenza testing performed during hospitalization or in 3 days prior to admission.

Data may include more than one hospitalization per woman across seasons; more than one hospitalization within a season will be addressed as follows:

- A second hospitalization (with an ARI/F code) admission that occurs ≥15 days from discharge date of a previous hospitalization with ARI/F will be considered a new hospitalization, adding to the denominator of observed hospital events for that season.
- A second hospitalization (with an ARI/F code) will be considered part of the same hospital event if the second admission occurs ≤14 days of the prior hospitalization discharge date. How information from both hospitalizations will be utilized may vary by manuscript project. For the VE project:
  - Any influenza testing that occurred during either hospitalization or within 3 days prior to either admission will be examined. RT-PCR influenza positive result from any of these tests will indicated an influenza positive hospitalization.
  - Reference both sets of discharge codes when creating derived variables. For example, ICD-10 code of J10 listed in the second set of discharge codes but not the first would still result in a “1” for ARIF_SUM_PI.

Different manuscript projects will utilize these events differently. For example, the vaccine effectiveness (VE) paper will exclude hospitalizations if they do not involve an ARI/F code even if influenza testing was performed.

The variables in Section XIII: Vaccination Patterns apply to the following population:

One record per pregnancy per influenza season (Unique PID-SEASON_YEAR combo). Each record meets all of the following criteria:

a) Female age 18-50 years as of pregnancy start date. Exclude any women that were less than 18 years or over 50 years as of pregnancy start date.
b) Pregnancy overlapped at least one of the pre-defined, site-specific influenza seasons.
c) Pregnancy reached >=20 weeks gestation

Notes: If a pregnancy overlaps two flu seasons, two records are generated, one for each season. If a woman has two pregnancies in a single influenza season, use the first pregnancy.

XV.

Variable Name	Variable Label	Variable Description	Value Labels	Notes
**II. Setting and Season Information**				
SITE	Sub-site or region	Region within a site – larger than the hospital-level	Site specific variable	
PID	Participant ID	Participant ID – uniquely associated with each individual like medic rec # or unique ID for your site	Site specific variable	Same woman can appear more than once in the dataset.
SEASON_YEAR	Season year of hospitalization	Each site to create variable to uniquely identify each season. Sites will have documented start and end of season in supplemental materials.	"2010/11"<br>"2011/12"<br>"2012/13"<br>"2013/14"<br>"2014/15"<br>"2015/16"	Each site will document the start and end date of each local influenza season per Network procedures
SEASON_FLU_PERIOD	Level of influenza circulation when hospitalized	Level of influenza circulation when hospitalized	1 = Early season with sustained local circulation at low levels<br>2 = High and peak periods of circulation<br>3 = Late season with sustained local circulation at low levels	Each site will document how each of these periods are defined per season in their supplemental material, using common cross-site procedures. Data set may include women enrolled outside of flu season for sub-studies such as focus on RSV.

SEASON_RSV_PERIOD	Hospitalization during or outside of defined RSV season	Hospitalization during or outside of defined RSV season	0 = Outside of RSV season<br>1 = Early season with sustained local circulation at low levels<br>2 = High and peak periods of circulation<br>3 = Late season with sustained local circulation at low levels	Start and end dates of RSV season are defined using common cross-site procedures that are consistent with the procedures for defining influenza season.

Variable Name	Variable Label	Variable Description	Value Labels	Notes
**III. Acute Respiratory or Febrile Illness (ARI/F) and Other Summary Variables from Discharge Codes (DC)**				
ARIF_SUM_DI	ARI/F dx at discharge dichotomy	Acute respiratory or febrile ICD dx at discharge	0 = None of ARI/F codes listed at discharge<br>1 = One or more ARI/F codes listed at discharge	Note that all events in the primary study analyses will have one of these codes. It is included as a variable for sites that plan to extract a broader set of hospital events that may not be ARI/F.<br><br>See Appendix of this document for ICD-10 code definitions.<br><br>ICD-9: 079.6, 079.99, 460-466, 478.19, 480-488, 490, 510.9, 511.0, 511.1, 511.89, 518.0, 518.4, 518.81, 519, 659.21, 669.11, 669.12, 669.14, 672.02, 672.04, 780.60, 780.64, 785.50, 785.52, 786.00, 786.02, 786.05, 786.09, 786.30, 786.05, 786.06, 786.07, 786.09, 786.1, 786.2, 786.4, 786.52, 786.7, 799.02, 799.1, 995.91, 995.92<br><br>ICD-10: A41.9, B34, B97.4, B97.8, J00-J04, J06, J09-J18, J20, J21, J22, J39.8, J39.9, J40, J80, J81, J86.9, J90, J96.0, J96.00-J96.02, J96.90-J96.92 (ICD-10-CA: J9690, J9691, J9699), J98.09 (ICD-10-CA: J980), J98.11 (ICD-10-CA: J9810), J98.19, J98.4, J98.8, J98.9, O75.1, O75.2 (ICD-10-CA: O75201, O75203, O75209), O86.4 (ICD-10-CA: O86402, O86404, O86409), O98.51-O98.53 (ICD-10-CA: O98501, O98502, O98504, O98509), O98.81, O98.811-O98.813, O98.819 (ICD-10-CA: O98801-O98804, O98809), O99.511-O99.513, O99.519 (ICD-10-CA: O99501-O99504, O99509), R04.2, R05, R06.0 (ICD-10-CA/AM: R060), R06.1, R06.2, R06.8, R06.82 (ICD-10-CA: R064), R07.1, R09.0, R09.02, R09.1, R09.2, R09.3, R09.81, R09.89 R50, R57.9, R65.21, R68.83 (ICD-10-CA: R680)
ARIF_SUM_PRIMARY	ARI/F code is primary diagnostic code	Indicates if one of the ARI/F codes listed in ARIF_SUM_DI above was the primary diagnosis or first listed	0 = ARI/F code not primary or first diagnosis<br>1 = ARI/F code was primary or first diagnosis<br>99 = No ARI/F code at discharge	
ARIF_SUM_PI	Pneumonia or influenza dx	Pneumonia or influenza listed in DC	0 = Neither pneumonia or influenza listed<br>1 = Either pneumonia and or influenza listed in DC	ICD-9: 480 – 487<br><br>ICD-10: J10 – J18, J09
ARIF_SUM_INFLU	Influenza dx	Influenza listed in DC	0 = No influenza code listed<br>1 = One or more influenza code listed in DC	ICD-9: 487<br><br>ICD-10: J09, J10, J11

Variable Name	Variable Label	Variable Description	Value Labels	Notes
ARIF_SUM_PNEUM	Pneumonia dx	Pneumonia listed in DC	0 = No pneumonia code listed<br>1 = One or more pneumonia code listed in DC	ICD-9: 480-486, 487.01<br><br>ICD-10: J12-J18
ARIF_SUM_FEBRILE	Febrile dx	Febrile dx listed in DC	O = No febrile code listed<br>1 = One or more febrile code listed	ICD-9: 659.21, 672.02, 672.04, 780.60, 780.64<br><br>ICD-10: O75.2 (ICD-10-CA: O75201, O75203, O75209), O86.4 (ICD-10-CA: O86402, O86404, O86409), R50, R68.83 (ICD-10-CA: R680)
ARIF_SUM_RESP	Acute respiratory diagnosis	Acute respiratory diagnosis at DC	0 = No ARI dx<br>1 = 1 or more ARI dx	Any of the following sub-variables is equal to 1:<br>ARIF_SUM_PNEUM<br>ARIF_RESP_FAIL_ARREST<br>ARIF_PULMON_COLL<br>ARIF_PLEUR_EFFSN<br>HR_ARDS<br>HR_ASTHMA_EXACER<br>HR_PNEUMOTHORAX<br>HR_LUNG_ABSC
HOSP_SUM_METABOLIC	Acute renal or liver failure	Acute renal or liver failure at DC	0 = None<br>1 = Acute renal or liver failure	Any of the following sub-variables is equal to 1:<br>HR_RENAL_FAIL<br>HR_LIVER_FAIL
HOSP_SUM_CARDIO	Acute cardiovascular event	Acute cardiovascular event at DC	0 = None<br>1 = Acute cardiovascular event	Any of the following sub-variables is equal to 1:<br>HR_HEART_FAIL<br>HR_ART_VEN_EMBOL<br>HR_PULM_EMBOL<br>HR_CARDITIS<br>HR_CARD_ARRST
HOSP_SUM_NEURO	Acute neurologic event	Acute neurologic event at DC	0 = None<br>1 = Acute neurologic event	Any of the following sub-variables is equal to 1:<br>HR_ALT_MENT<br>HR_ENCEPHAL<br>HR_MENINGITIS<br>HR_ANOX_BRN_DMG<br>HR_ENCEPH_MYEL<br>HR_COMA<br>HR_EPILEP<br>HR_POLYNEURO

Variable Name	Variable Label	Variable Description	Value Labels	Notes
HOSP_SUM_MUSC	Acute musculoskeletal event	Acute musculoskeletal event at DC	0 = None<br>1 = Acute musculoskeletal event	Any of the following sub-variables is equal to 1:<br>HR_RHABDOMYOL<br>HR_MYOSITIS<br>HR_MYOPATHY
HOSP_SUM_CEREBRO	Acute cerebrovascular event	Acute cerebrovascular event at DC	0 = none<br>1 = Acute cerebrovascular event	Any of the following sub-variables is equal to 1:<br>HR_CEREBRAL_INFARC<br>HR_CEREBRAL_HEMR<br>HR_CEREBRAL_EDEMA
HOSP_SUM_ENDOCRINE	Acute endocrine event	Acute endocrine event at DC	0 = None<br>1 = Acute endocrine event	Any of the following sub-variables is equal to 1:<br>HR_KETAOACIDOSIS<br>HR_HYPRSMOLRTY<br>HR_DIABET_COMA<br>HR_THYRD_STORM
HOSP_SUM_VOM_HYPER	Excessive vomiting complication	Excessive vomiting complication at DC	0 = None<br>1 = Excessive vomiting complication	PC_VOMIT is equal to 1
HOSP_SUM_ANY_CRIT	Any critical illness	Any critical illness at DC	0 = None<br>1 = Critical illness	Any of the following sub-variables is equal to 1:<br>ICU_ANY<br>ARIF_RESP_FAIL_ARREST<br>HOSP_ECMO<br><br>Or HOSP_DCSTATUS is equal to 4.

Variable Name	Variable Label	Variable Description	Value Labels	Notes
**IV. Acute Respiratory or Febrile Illness (ARI/F) Specific Diagnosis Codes**				
ARIF_RSV	RSV dx at discharge	RSV dx at discharge	0 = No code listed at discharge<br>1 = One or more codes listed at discharge	ICD-9: 079.6<br><br>ICD-10: B97.4
ARIF_VIRAL_UNSPEC	Unspecified viral infection dx at discharge	Unspecified viral infection dx at discharge	0 = No code listed at discharge<br>1 = One or more codes listed at discharge	ICD-9: 079.99<br><br>ICD-10:B34, B97.8

ARIF_NASOPHAR	Acute nasopharyngitis (common cold) dx at discharge	Acute nasopharyngitis (common cold) dx at discharge	0 = No code listed at discharge<br>1 = One or more codes listed at discharge	ICD-9: 460<br><br>ICD-10: J00
ARIF_SINUS	Acute sinusitis dx at discharge	Acute sinusitis dx at discharge	0 = No code listed at discharge<br>1 = One or more codes listed at discharge	ICD-9: 461<br><br>ICD-10: J01
ARIF_PHARYN	Acute pharyngitis dx at discharge	Acute pharyngitis dx at discharge	0 = No code listed at discharge<br>1 = One or more codes listed at discharge	ICD-9: 462<br><br>ICD-10: J02
ARIF_TONSIL	Acute tonsillitis dx at discharge	Acute tonsillitis dx at discharge	0 = No code listed at discharge<br>1 = One or more codes listed at discharge	ICD-9: 463<br><br>ICD-10: J03
ARIF_LARYN	Acute laryngitis and tracheitis dx at discharge	Acute laryngitis and tracheitis dx at discharge	0 = No code listed at discharge<br>1 = One or more codes listed at discharge	ICD-9: 464<br><br>ICD-10: J04
ARIF_MULTARI	Acute upper respiratory infections of multiple or unspecified sites dx at discharge	Acute upper respiratory infections of multiple or unspecified sites dx at discharge	0 = No code listed at discharge<br>1 = One or more codes listed at discharge	ICD-9: 465<br><br>ICD-10: J06
ARIF_BRONCH	Acute bronchitis and bronchiolitis dx at discharge	Acute bronchitis and bronchiolitis dx at discharge	0 = No code listed at discharge<br>1 = One or more codes listed at discharge	ICD-9: 466<br><br>ICD-10: J20, J21

ARIF_PNEUMON_VIRAL	Viral pneumonia dx at discharge	Viral pneumonia dx at discharge	0 = No code listed at discharge<br>1 = One or more codes listed at discharge	ICD-9: 480<br><br>ICD-10: J12
ARIF_PNEUMOCOCCAL	Pneumococcal pneumonia dx at discharge	Pneumococcal pneumonia dx at discharge	0 = No code listed at discharge<br>1 = One or more codes listed at discharge	ICD-9: 481<br><br>ICD-10: J13, J14, J15
ARIF_PNEUMON_OTHERBAC	Other bacterial pneumonia dx at discharge	Other bacterial pneumonia dx at discharge	0 = No code listed at discharge<br>1 = One or more codes listed at discharge	ICD-9: 482
ARIF_PNEUMON_OTHER_SPEC	Pneumonia due to other specified organism dx at discharge	Pneumonia due to other specified organism dx at discharge	0 = No code listed at discharge<br>1 = One or more codes listed at discharge	ICD-9: 483<br><br>ICD-10: J16
ARIF_PNEUMON_ID	Pneumonia in infectious diseases classified elsewhere dx at discharge	Pneumonia in infectious diseases classified elsewhere dx at discharge	0 = No code listed at discharge<br>1 = One or more codes listed at discharge	ICD-9: 484<br><br>ICD-10: J17
ARIF_BRONCHPNEUMON	Bronchopneumonia, organism unspecified dx at discharge	Bronchopneumonia, organism unspecified dx at discharge	0 = No code listed at discharge<br>1 = One or more codes listed at discharge	ICD-9: 485<br><br>ICD-10: J18
ARIF_PNEUMON_UNSPEC	Pneumonia, organism unspecified dx at discharge	Pneumonia, organism unspecified dx at discharge	0 = No code listed at discharge<br>1 = One or more codes listed at discharge	ICD-9: 486

ARIF_INFLU	Influenza dx at discharge	Influenza dx at discharge	0 = No code listed at discharge<br>1 = One or more codes listed at discharge	ICD-9: 487<br><br>ICD-10: J10, J11
ARIF_INFLUA	Influenza due to identified novel influenza A virus dx at discharge	Influenza due to identified novel influenza A virus dx at discharge	0 = No code listed at discharge<br>1 = One or more codes listed at discharge	ICD-9: 488.01-488.89<br><br>ICD-10: J09
ARIF_BRONCH_UNSPEC	Bronchitis, unspecified dx at discharge	Bronchitis, unspecified dx at discharge	0 = No code listed at discharge<br>1 = One or more codes listed at discharge	ICD-9: 490<br><br>ICD-10: J40
ARIF_PULMON_COLL	Pulmonary collapse dx at discharge	Pulmonary collapse dx at discharge	0 = No code listed at discharge<br>1 = One or more codes listed at discharge	ICD-9: 518.0<br><br>ICD-10: J98.19
ARIF_PULMON_EDEMA	Pulmonary edema dx at discharge	Pulmonary edema dx at discharge	0 = No code listed at discharge<br>1 = One or more codes listed at discharge	ICD-9: 518.4<br><br>ICD-10: J81
ARIF_RESP_FAIL_ARREST	Acute respiratory failure or arrest dx at discharge	Acute respiratory failure or arrest dx at discharge	0 = No code listed at discharge<br>1 = One or more codes listed at discharge	ICD-9: 518.81, 799.1<br><br>ICD-10: J96.0, J96.00-J96.02, J96.2, J96.90-J96.92, R09.2<br>ICD-10-CA: J9600, J9601, J9690, J9691, J9699
ARIF_RESP_UNSPEC	Unspecified disease of respiratory system dx at discharge	Unspecified disease of respiratory system dx at discharge	0 = No code listed at discharge<br>1 = One or more codes listed at discharge	ICD-9: 519<br><br>ICD-10: J22, J39.8, J39.9, J98.09, J98.4, J98.8, J98.9<br>ICD-10-CA: J980

ARIF_FEVER	Fever, unspecified dx at discharge	Fever, unspecified dx at discharge	0 = No code listed at discharge<br>1 = One or more codes listed at discharge	ICD-9: 780.60<br><br>ICD-10: R50
ARIF_CHILLS	Chills (without fever) dx at discharge	Chills (without fever) dx at discharge	0 = No code listed at discharge<br>1 = One or more codes listed at discharge	ICD-9: 780.64<br><br>ICD-10: R68.83 (does not exist to specificity in ICD-10-AM)<br>ICD-10-CA: R680
ARIF_RESP_ABNORM	Respiratory abnormality, unspecified dx at discharge	Respiratory abnormality, unspecified dx at discharge	0 = No code listed at discharge<br>1 = One or more codes listed at discharge	ICD-9: 786.00<br><br>ICD-10: J98.9<br>ICD-10-AM: Does not exist (maps to R06.8)
ARIF_SHORT_BREATH	Shortness of breath dx at discharge	Shortness of breath dx at discharge	0 = No code listed at discharge<br>1 = One or more codes listed at discharge	ICD-9: 786.05<br><br>ICD-10: R06.02<br>ICD-10-CA: R060<br>ICD-10-AM: R06.0
ARIF_TACHYP	Tachypnea dx at discharge	Tachypnea dx at discharge	0 = No code listed at discharge<br>1 = One or more codes listed at discharge	ICD-9: 786.06<br><br>ICD-10: R06.82<br>ICD-10-CA: R064<br>ICD-10-AM: R06.8
ARIF_WHEEZE	Wheezing dx at discharge	Wheezing dx at discharge	0 = No code listed at discharge<br>1 = One or more codes listed at discharge	ICD-9: 786.07<br><br>ICD-10: R06.2
ARIF_RESP_OTHER	Respiratory abnormality, other dx at discharge	Respiratory abnormality, other dx at discharge	0 = No code listed at discharge<br>1 = One or more codes listed at discharge	ICD-9: 786.09

ARIF_STRIDOR	Stridor dx at discharge	Stridor dx at discharge	0 = No code listed at discharge<br>1 = One or more codes listed at discharge	ICD-9: 786.1<br><br>ICD-10: R06.1
ARIF_COUGH	Cough dx at discharge	Cough dx at discharge	0 = No code listed at discharge<br>1 = One or more codes listed at discharge	ICD-9: 786.2<br><br>ICD-10: R05
ARIF_SPUTUM	Abnormal sputum dx at discharge	Abnormal sputum dx at discharge	0 = No code listed at discharge<br>1 = One or more codes listed at discharge	ICD-9: 786.4<br><br>ICD-10: R09.3
ARIF_RESP_PAIN	Painful respiration dx at discharge	Painful respiration dx at discharge	0 = No code listed at discharge<br>1 = One or more codes listed at discharge	ICD-9: 786.52<br><br>ICD-10: R07.1
ARIF_SOUND	Abnormal chest sounds dx at discharge	Abnormal chest sounds dx at discharge	0 = No code listed at discharge<br>1 = One or more codes listed at discharge	ICD-9: 786.7<br><br>ICD-10: R09.89
ARIF_HYPOX	Hypoxemia dx at discharge	Hypoxemia dx at discharge	0 = No code listed at discharge<br>1 = One or more codes listed at discharge	ICD-9: 799.02<br><br>ICD-10: R09.02<br>ICD-10-AM: R09.0
ARIF_RDS	Acute respiratory distress syndrome dx at discharge	Acute respiratory distress syndrome dx at discharge	0 = No code listed at discharge<br>1 = One or more codes listed at discharge	ICD-10: J80
ARIF_ATELECT	Atelectasis dx at discharge	Atelectasis dx at discharge	0 = No code listed at discharge<br>1 = One or more codes listed at discharge	ICD-10: J98.11<br>ICD-10-CA: J9810

ARIF_RESP_PC	Diseases of the respiratory system complicating pregnancy dx at discharge	Diseases of the respiratory system complicating pregnancy dx at discharge	0 = No code listed at discharge<br>1 = One or more codes listed at discharge	ICD-10: O99.511, O99.512, O99.513, O99.519<br>ICD-10-CA: O99501, O99502, O99503, O99504, O99509<br>ICD-10-AM: O99.5
ARIF_PYREX_LABOR	Pyrexia during labor dx at discharge	Pyrexia during labor dx at discharge	0 = No code listed at discharge<br>1 = One or more codes listed at discharge	ICD-9: 659.21<br><br>ICD-10: O75.2<br>ICD-10-CA: O75201, O75203, O75209
ARIF_PYREX_DELIV	Pyrexia following delivery dx at discharge	Pyrexia following delivery dx at discharge	0 = No code listed at discharge<br>1 = One or more codes listed at discharge	ICD-9: 672.02, 672.04<br><br>ICD-10: O86.4<br>ICD-10-CA: O86402, O86404, O86409
ARIF_ID_MAT	Other maternal infectious and parasitic diseases complicating pregnancy	Other maternal infectious and parasitic diseases complicating pregnancy	0 = No code listed at discharge<br>1 = One or more codes listed at discharge	ICD-10: O98.81, O98.811-O98.813, O98.819<br>ICD-10-CA: O98801-O98804, O98809
ARIF_VIRAL_MAT	Other viral diseases complicating pregnancy	Other viral diseases complicating pregnancy	0 = No code listed at discharge<br>1 = One or more codes listed at discharge	ICD-10: O98.51-O98.53<br>(ICD-10-CA: O98501-O98504, O98509
ARIF_PLEUR_EFFSN	Pleural effusion, not elsewhere classified	Pleural effusion, not elsewhere classified	0 = No code listed at discharge<br>1 = One or more codes listed at discharge	ICD-9: 510.9, 511.0, 511.1, 511.89<br><br>ICD-10: J86.9, J90, R09.1
ARIF_HEMTYS	Hemoptysis	Hemoptysis	0 = No code listed at discharge<br>1 = One or more codes listed at discharge	ICD-9: 786.30<br><br>ICD-10: R04.2
ARIF_DYSPNEA	Dyspnea	Dyspnea	0 = No code listed at discharge<br>1 = One or more codes listed at discharge	ICD-9: 786.02, 786.05, 786.09<br><br>ICD-10: R06.0
ARIF_CONGEST	Nasal congestion	Nasal congestion	0 = No code listed at discharge<br>1 = One or more codes listed at discharge	ICD-9: 478.19<br><br>ICD-10: R09.81

ARIF_SEPSIS	Sepsis	Sepsis	0 = No code listed at discharge<br>1 = One or more codes listed at discharge	ICD-9: 669.11, 669.12, 669.14, 785.50, 785.52, 995.91, 995.92<br><br>ICD-10: A41.9, R57.9, R65.21, O75.1

Variable Name	Variable Label	Variable Description	Value Labels	Notes
**V. Maternal Characteristics**				
MAT_DOB	Maternal DOB			
MAT_AGE_ADM	Maternal Age at Admission	Date of admission minus DOB: ADM_DT minu MAT_DOB		Age in years (whole number)
MAT_AGE_35	Maternal Age at Admission LT or GE 35 years	Maternal Age at Admission LT or GE 35 years	1 = < 35 years<br>2 = >=35 years<br>99 = Unknown	
MAT_RACE	Maternal race		1 = Caucasian<br>2 = Other<br>99 = Unknown	Each site defines additional categories other than Caucasian; common variables limited to "Caucasian" and "Other."
MAT_REGION	Population density of maternal region of residence	Woman resides in either urban or rural region	1 = Urban<br>2 = Rural<br>99 = Unknown	Use country-specific classification of urban vs. rural
MAT_ENROLL	Maternal eligibility for sample	Woman member of health plan or population for at least 12 months	1 = Enrolled/eligible for >= 12 months prior to pregnancy start date<br>2 = Enrolled/eligible for < 12 months prior to pregnancy start date<br>99 = Unknown	Each site to define whether enrollment needs to be continuous across the 12-month period.
MAT_SES	Maternal SES site categories	SES using site categories	1 = Not low SES<br>2 = Low SES<br>99 = Unknown	Optional – for sites who have data available. Sites should define variable for their site, e.g. Medicaid vs. not, deprivation index.
MAT_SMOKE_PREG	Maternal smoking documented at any time during pregnancy		0 = No, smoking during pregnancy documented<br>1 = Yes, smoking during pregnancy documented<br>99 = Unknown	Optional – for sites who have data available. This is a characteristic of pregnancy that is not limited to the index hospitalization. Sites should document method used to collect.

MAT_SMOKE_PRIOR	Maternal smoking documented prior to pregnancy	Maternal smoking documented within ~2 years prior to pregnancy	1 = Yes, smoking documented prior to pregnancy<br>2 = No, smoking not documented<br>99 = unknown	
MAT_HEIGHT	Height measurement prior to pregnancy	Height recorded since aged >17 years old	Centimeters (cm)	Optional – for sites who have data available
MAT_WEIGHT_PRIOR	Weight measurement prior to pregnancy	Weight recorded within ~2 years prior to pregnancy; most recent weight prior to pregnancy	Kilograms (kg)	Optional – for sites who have data available
MAT_BMI_PRIOR	BMI prior to pregnancy	Pre-pregnancy BMI documented or calculated from measurements; most recent weight prior to pregnancy		Optional – for sites who have data available
MAT_BMI_PRIOR_OBESE	Obese prior to pregnancy by BMI	Pre-pregnancy BMI meets standard definition of obesity	1 = Obese (BMI >= 30)<br>0 = Not obese (BMI < 30)<br>99 = Unknown	Optional – for sites who have data available<br><br>WHO standard at http://www.who.int/mediacentre/factsheets/fs311/en/

Variable Name	Variable Label	Variable Description	Value Labels	Notes
**VI. High Risk Medical Conditions in Discharge Codes**				
HR_DI	High risk medical conditions identified at index hospitalization dichotomy	High risk medical condition (chronic condition, not pregnancy complication) identified from discharge code of index hospital admission	0 = No high risk medical condition (not pregnancy complication) documented at index hospitalization<br>1 = One or more high risk medical condition(s) documented at index hospitalization	See Appendix of this document for ICD-10 code definitions.<br><br>Sites may include additional variables if they are able to also collect high risk conditions for one-year prior to admission from any encounters, ambulatory or inpatient.
HR_DI_EXCLD_ANEMIA	High risk medical conditions identified at index hospitalization dichotomy EXCEPT ANEMIA	High risk medical condition (chronic condition, not pregnancy complication) identified from discharge code of index hospital admission EXCEPT ANEMIA	0 = No high risk medical condition (not pregnancy complication) documented at index hospitalization, OTHER THAN ANEMIA<br>1 = One or more high risk medical condition(s) documented at index hospitalization, OTHER THAN ANEMIA	HR_DI_EXCLD_ANEMIA = (HR_DI) – (HR_BLOOD) + (HR_BLOOD_EXCLD_ANEMIA)

HR_BLOOD	Blood disorder dx identified at index hospitalization	Blood disorder dx, identified from discharge code of index hospitalization	0 = No blood disorder dx documented<br>1 = Blood disorder dx	ICD-9: 280, 281, 282, 283, 284, 285.0, 285.21, 285.22, 285.29, 285.3, 285.8, 285.9, 286, 287, 288, 288.0-288.2, 289<br><br>ICD-10: D50.0, D50.8, D50.9, D51, D52, D53, D58, D59, D61, D64.0-3, D63.1, D63.0, D63.8, D64.81, D64.89, D64.9, D68, D69, D70, D71, D72, D73, D74, D75, D77<br><br>ICD-10-AM: D63.1 does not exist. D63.8 existed until July 2010. D63* is assigned for anaemia in specific conditions for which chronic kidney disease is not one. Both anaemia and CKD would be coded separately. D63.0 does not exist after July 2010. Post July 2010 the anaemia and neoplastic disease are coded separately [D63.0 until July 2010 and then D64.9 and C00-D48)]. D63.8 does not exist post July 2010. D63 is coded for anaemia in specific conditions only. [D63.8 until July 2010 then D63 with specific conditions]. D64.8 is other specified anaemias, D64.89 does not exist. Chronic lymphadenitis 289.1 maps to I88.1, 289.2 maps to I88.0, 289.3 maps to I88.9.
HR_BLOOD_EXCLD_ANEMIA	Blood disorder dx identified at index hospitalization OTHER THAN ANEMIA	Blood disorder dx, identified from discharge code of index hospitalization OTHER THAN ANEMIA	0 = No blood disorder dx documented OTHER THAN ANEMIA<br>1 = Blood disorder dx OTHER THAN ANEMIA	ICD-9: 282, 283, 284, 285.0, 285.21, 285.22, 285.29, 285.3, 286, 287, 288, 288.0-288.2, 289<br><br>ICD-10: D58, D59, D61, D64.0-3, D63.1, D63.0, D63.8, D64.81, D68, D69, D70, D71, D72, D73, D74, D75, D77<br><br>ICD-10-AM: Also include D53
HR_DIABT	Diabetes dx identified at index hospitalization	Diabetes dx, identified from discharge code of index hospitalization	0 = No diabetes dx documented<br>1 = Diabetes dx	ICD-9: 249, 250, 357.2, 362.0, 366.41<br><br>ICD-10: E08-E11<br><br>ICD-10-AM: E08 does not exist. E13.x other specified diabetes i.e. drug induced. Diabetes mellitus is classified to type 1 and type 2, intermediate hyperglycaemia, other specified and unspecified [E09, E10, E11, E12, E13, E14].

HR_HEART_FAIL	Acute heart failure dx identified at index hospitalization	Acute heart failure dx identified at index hospitalization	0 = No acute heart failure dx documented<br>1 = Acute heart failure dx	ICD-9: 428.21, 428.23, 428.31, 428.33, 428.41, 428.43<br><br>ICD-10: I50.21, I50.23, I50.31, I50.33, I50.41, I50.43<br><br>ICD-10-AM: I50 (do not have codes to specificity)
HR_CARD_ARRST	Cardiac arrest dx identified at index hospitalization	Cardiac arrest dx identified at index hospitalization	0 = No cardiac arrest dx documented<br>1 = Cardiac arrest dx	ICD-9: 427.5<br><br>ICD-10: I46.9
HR_HEART	Heart disease dx identified at index hospitalization	Heart disease dx, identified from discharge code of index hospitalization	0 = No heart/cardiac disease dx documented<br>1 = Heart/cardiac disease dx	ICD-9: 093, 112.81, 130.3, 362.11, 391, 393, 394, 395, 396, 397, 398, 402, 404, 410, 411, 412, 413, 414, 415, 416, 417, 421, 423, 424, 425, 427.1-427.5, 427.8, 427.9, 428, 429, 440, 441, 442, 443, 444, 445, 446, 447,745, 746, 747, 996.0, 996.1, V43.3, V45.0, V45.81, V45.82<br><br>ICD-10: H35.031-039, I01, I09.2, I05, I06, I34, I35, I36, I37, I09, I11, I12, I13, I21, I24, I25.2, I20, I25.700-I25.799, I26, I27, I28, I33, I30, I31, I38, I39, I42, I46, I47, I48, I49, I50, I40, I41, I51, I52, I70, I71, I72, I73, I74, I75 (ICD-10-AM: I70.9), M30.0, M31, I77, Q21, Q22, Q23, Q24, Q25, Q26, Q27, T82, Z95
HR_CANCER	Cancer dx identified at index hospitalization	Cancer dx, identified from discharge code of index hospitalization	0 = No cancer dx documented<br>1 = Cancer dx	ICD-9: 140, 141, 142, 143, 144, 145, 146, 147, 148, 149, 150, 151, 152, 153, 154, 155, 156, 157, 158, 159, 160, 161, 162, 163, 164, 165, 170, 171, 172, 174, 175, 176, 179, 180, 181, 182, 183, 184, 185, 186, 187, 188, 189, 190, 191, 192, 193, 194, 195, 196, 197, 198, 199, 200, 201, 202, 203, 204, 205, 206, 207, 208, 209, 231.0-231.2, 231.8-231.9, 235-238, V58.0-V58.1<br><br>ICD-10: C00, C01, C02, C07, C08, C03, C04, C05, C06, C09, C10, C11, C12, C13, C14, C15, C16, C17, C18, C19, C20, C21, C22, C23, C24, C25, C48, C26, C30, C31, C32, C33, C34, C38, C37, C39, C40, C41, C44, C49, C43, C50, C46, C55, C53, C58, C54, C56, C51, C52, C61, C62, C60, C67, C64, C69, C71, C72, C73, C74, C75, C76, C77, C78, C79, C80, C81, C83, C90, C91, C92, C93, C94, C95, C7A (does not exist in ICD-10-AM), D02, D37-44, D48, Z51.11, Z51.12
HR_IMMUNE	Immune disorder dx identified at index hospitalization	Immune disorder dx, identified from discharge code of index hospitalization	0 = No immune disorder dx documented<br>1 = Immune disorder dx	ICD-9: 042, 079.5, 136.3, 279, 289.4, 289.5, 358.3, 710, 714, 795, 960.7, 963.1, V08, V42.0-V42.2, V42.6-V42.9<br><br>ICD-10: B20, B33.3, B59, B80, B81, B82, B73.1, D73, G70.8, M32.10, M35, M05, M06, R82.8, R84.7, R85.7, R86.7, R87.7, R89.7, T45 (ICD-10-AM: T45.1), Z21, Z94

HR_RENAL_FAIL	Acute renal failure dx identified at index hospitalization	Acute renal failure dx identified at index hospitalization	0 = No acute renal failure dx documented<br>1 = Acute renal failure dx	ICD-9: 404.02, 404.03, 572.4, 584<br><br>ICD-10: I13.11, I13.2, K76.7, N17
HR_RENAL	Renal disease dx identified at index hospitalization	Renal disease dx, identified from discharge code of index hospitalization	0 = No renal disease dx documented<br>1 = Renal disease dx	ICD-9: 285.21, 403, 581, 582, 583, 584, 585, 586, 587, 588.0, 588.1, 589, 593.8, 753.1, 996.56, 996.73, V56, V45.1<br><br>ICD-10: D63.1 (does not exist in ICD-10-AM), I12.0, I13.11 (does not exist to specificity in ICD-10-AM), I13.2, N04, N03, N05, N17, N18, N19, N26.9 (does not exist to specificity in ICD-10-AM), N25.0, N25.1, N27.0, N27.1, N27.9, N28.89 (does not exist to specificity in ICD-10-AM), Q61, T85, Z49, Z91.15 (does not exist to specificity in ICD-10-AM)
HR_LIVER_FAIL	Acute liver failure dx identified at index hospitalization	Acute liver failure dx identified at index hospitalization	0 = No acute liver failure dx documented<br>1 = Acute liver failure dx	ICD-9: 570, 573.4<br><br>ICD-10: K72.00, K76.3, K76.2
HR_LIVER	Liver disease dx identified at index hospitalization	Liver disease dx, identified from discharge code of index hospitalization	0 = No liver disease dx documented<br>1 = Liver disease dx	ICD-9: 571, 572.1-572.8<br><br>ICD-10: K70, K73, K74, K75.1-K75.9
HR_ASTHMA	Asthma dx identified at index hospitalization	Asthma dx, identified from discharge code of index hospitalization	0 = No asthma dx documented<br>1 = Asthma dx	ICD-9: 493<br><br>ICD-10: J45
HR_ASTHMA_EXACER	Asthma exacerbation dx identified at index hospitalization	Asthma exacerbation dx identified at index hospitalization	0 = No asthma exacerbation dx documented<br>1 = Asthma exacerbation dx	ICD-9: 493.01, 493.02, 493.11, 493.12, 493.21, 493.22, 493.91, 493.92<br><br>ICD-10: J44.0, J44.1, J45.21, J45.22, J45.901, J45.902
HR_ARDS	Other pulmonary insufficiency not elsewhere classified dx identified at index hospitalization	Other pulmonary insufficiency not elsewhere classified dx identified at index hospitalization	0 = No ARDS dx documented<br>1 = ARDS dx	ICD-9: 518.82<br><br>ICD-10: J80
HR_PNEUMOTHORAX	Pneumothorax dx identified at index hospitalization	Pneumothorax dx identified at index hospitalization	0 = No pneumothorax dx documented<br>1 = Pneumothorax dx	ICD-9: 512<br><br>ICD-10: J93
HR_LUNG_ABSC	Lung abscess dx identified at index hospitalization	Lung abscess dx identified at index hospitalization	0 = No lung abscess dx documented<br>1 = Lung abscess dx	ICD-9: 513<br><br>ICD-10: J85

HR_OLUNG	Lung disease dx not asthma at index hospitalization	Lung disease dx, not asthma identified from discharge code of index hospitalization	0 = No lung disease dx documented<br>1 = Lung disease dx	ICD-9: 010, 011, 012, 018, 031.0, 135, 137.0, 277, 490, 491, 492, 494, 495, 496, 500, 501, 502, 503, 504, 505, 506, 507.0, 507.1, 507.8, 508, 510, 511, 512, 513, 514, 515, 516, 517, 518, 519, 748.4-748.6<br><br>ICD-10: A15, A17, A18, A19, A31, D86, E84, J40, J41, J42, J43, J47, J60, J61, J62.0, J62.8, J63.6 (ICD-10-AM: J63.8), J64, J67, J68, J69.0, J69.1, J69.8, J70.9, J85, J93, J98, M34, M35, Q34, R09.1
HR_OBESE	Obesity dx identified at index hospitalization	Obesity dx identified from discharge code of index hospitalization	0 = No obesity dx documented<br>1 = Obesity dx	ICD-9: 649.1, 278.00, 278.01, 278.03<br><br>ICD-10: E66, O99.210-215, O99.840-845<br>ICD-10-AM: O99.210-215, O99.840-845 does not exist
HR_OMETBOL	Metabolic disorder not obesity dx identified at index hospitalization	Metabolic disorder dx not obesity, identified from discharge code of index hospitalization	0 = No metabolic disorder dx documented<br>1 = Metabolic disorder dx	ICD-9: 240-246, 253, 254, 255, 260-269, 270, 271, 272, 273, 274, 275, 277.0-9<br><br>ICD-10: E03-E07, E22, E23, E32, E27, E40-E46, E50-E56, E70-E72, E74, E78, E88, M10, E83
HR_ALT_MENT	Altered mental status dx identified at index hospitalization	Altered mental status dx, identified from discharge code of index hospitalization	0 = No altered mental status dx documented<br>1 = Altered mental status dx	ICD-9: 293, 437.7, 780.02, 780.09, 780.1, 780.97<br><br>ICD-10: F05, F06.0, F06.1, F06.2, F06.30, F06.4, F06.8, G45.4, R40.0, R40.4, R41.82, R44.0
HR_ENCEPHAL	Encephalopathy dx identified at index hospitalization	Encephalopathy dx, identified from discharge code of index hospitalization	0 = No encephalopathy dx documented<br>1 = Encephalopathy dx	ICD-9: 348.3, 437.2, 572.2<br><br>ICD-10: G93.4, I67.4, K72.90, K72.91
HR_MENINGITIS	Meningitis dx identified at index hospitalization	Meningitis dx, identified from discharge code of index hospitalization	0 = No meningitis dx documented<br>1 = Meningitis dx	ICD-9: 003.21, 036, 047, 049.1, 112.83, 114.2, 320, 321, 322<br><br>ICD-10: A02.21, A39, A87.0, A87.1, B37.5, B38.4, G00, G01, G02, G03
HR_ANOX_BRN_DMG	Anoxic brain damage dx identified at index hospitalization	Anoxic brain damage dx, identified from discharge code of index hospitalization	0 = No anoxic brain damage dx documented<br>1 = Anoxic brain damage dx	ICD-9: 348.1<br><br>ICD-10: G93.1
HR_ENCEPH_MYEL	Encephalitis or myelitis dx identified at index hospitalization	Encephalitis or myelitis dx, identified from discharge code of index hospitalization	0 = No encephalitis or myelitis dx documented<br>1 = Encephalitis or myelitis dx	ICD-9: 036.1, 323, 341.2<br><br>ICD-10: A39.81, G05, G37.3

HR_COMA	Coma dx identified at index hospitalization	Coma dx, identified from discharge code of index hospitalization	0 = No coma dx documented<br>1 = Coma dx	ICD-9: 780.01, 780.03<br><br>ICD-10: R40.20, R40.3
HR_EPILEP	Status epilepticus dx identified at index hospitalization	Status epilepticus dx, identified from discharge code of index hospitalization	0 = No status epilepticus dx documented<br>1 = Status epilepticus dx	ICD-9: 345.2, 345.3<br><br>ICD-10: G40.A01, G40.A09, G40.A11, G40.A19, G40.301
HR_POLYNEURO	Critical illness polyneuropathy dx identified at index hospitalization	Critical illness polyneuropathy dx, identified from discharge code of index hospitalization	0 = No critical illness polyneuropathy dx documented<br>1 = Critical illness polyneuropathy dx	ICD-9: 357.82<br><br>ICD-10: G62.81
HR_CEREBRAL_INFARC	Cerebral infarction dx identified at index hospitalization	Cerebral infarction dx, identified from discharge code of index hospitalization	0 = No cerebral infarction dx documented<br>1 = Cerebral infarction dx	ICD-9: 346.6, 433.01, 433.11, 433.21, 433.31, 433.81, 433.91, 434.01, 434.11, 434.91<br><br>ICD-10: G43.6, I63.019, I63.119, I63.139, I63.20, I63.219, I63.22, I63.239, I63.30, I63.40, I63.50, I63.59
HR_CEREBRAL_HEMR	Cerebral hemorrhage dx identified at index hospitalization	Cerebral hemorrhage dx, identified from discharge code of index hospitalization	0 = No cerebral hemorrhage dx documented<br>1 = Cerebral hemorrhage dx	ICD-9: 430, 431, 432<br><br>ICD-10: I60.9, I61.9, I62
HR_CEREBRAL_EDEMA	Cerebral edema dx identified at index hospitalization	Cerebral edema dx, identified from discharge code of index hospitalization	0 = No cerebral edema dx documented<br>1 = Cerebral edema dx	ICD-9: 348.5<br><br>ICD-10: G93.6
HR_NEURO	Neurologic disease dx identified at index hospitalization	Neurologic disease dx, identified from discharge code of index hospitalization	0 = No neurologic disease dx documented<br>1 = Neurologic disease dx	ICD-9: 013, 046, 137.1, 290, 294.1, 310, 318, 330, 331, 332, 333, 334, 335, 336, 337, 340, 341, 342, 343, 344, 345,348, 349.1-9, 392, 430, 431, 432, 433, 434, 435, 436, 437, 438, 756.4, 780.3, 907.2, 996.2, 996.75<br><br>ICD-10: A17, A81, B90.0, F01, F02, F03, F07, F71, F72, F73, E75, G20, G25, G26, G11, M47, G60, G35, G36, G81, G80, G82, G40, G93, G96, I02, I60, I61, I62, I65, I66, I67.82 (ICD-10-AM: G45.9), I67.9, I69, G71.13 (does not exist to specificity in ICD-10-AM), R56, T85 (ICD-10-AM: T85.1)
HR_ART_VEN_EMBOL	Arterial or venous embolism dx identified at index hospitalization	Arterial or venous embolism dx identified at index hospitalization	0 = No arterial or venous embolism dx documented<br>1 = Arterial or venous embolism dx	ICD-9: 444, 449, 453<br><br>ICD-10: I74, I76, I82

HR_PULM_EMBOL	Pulmonary embolism dx identified at index hospitalization	Pulmonary embolism dx identified at index hospitalization	0 = No pulmonary embolism dx documented<br>1 = Pulmonary embolism dx	ICD-9: 415.1<br><br>ICD-10: I26
HR_CARDITIS	Carditis dx identified at index hospitalization	Carditis dx identified at index hospitalization	0 = No carditis dx documented<br>1 = Carditis dx	ICD-9: 112.81, 391, 420, 421, 422, 429<br><br>ICD-10: A39.50, B37.6, I01.0, I01.1, I01.2, I30, I33, I40, I51.4
HR_DIC	Defibrination syndrome dx identified at index hospitalization	Defibrination syndrome dx identified at index hospitalization	0 = No defibrination syndrome dx documented<br>1 = Defibrination syndrome dx	ICD-9: 286.6<br><br>ICD-10: D65
HR_CARDIOPULM	Any cardiopulmonary disease at index hospitalization		0 = No heart or lung code at index hospitalization<br>1 = One more heart or lung medical condition documented at index hospitalization	Any HEART, LUNG, ASTHMA, or ASTHMA_EXACER code
HR_KETOACIDOSIS	Ketoacidosis dx identified at index hospitalization	Ketoacidosis dx, identified from discharge code of index hospitalization	0 = No ketoacidosis dx documented<br>1 = Ketoacidosis dx	ICD-9: 249.1, 250.1<br><br>ICD-10: E08.1, E10.1, E13.1
HR_HYPRSMOLRTY	Hyperosmolarity dx identified at index hospitalization	Hyperosmolarity dx, identified from discharge code of index hospitalization	0 = No hypersmolarity dx documented<br>1 = Hypersmolarity dx	ICD-9: 249.2, 250.2<br><br>ICD-10: E08.0, E11.0, E13.0
HR_DIABET_COMA	Diabetic coma dx identified at index hospitalization	Diabetic coma dx, identified from discharge code of index hospitalization	0 = No diabetic coma dx documented<br>1 = Diabetic coma dx	ICD-9: 249.3, 250.3, 251<br><br>ICD-10: E08.11, E08.641, E09.11, E09.641, E10.11, E11.01, E11.641, E13.11, E13.641, E15
HR_THYRD_STORM	Thyroid storm dx identified at index hospitalization	Thyroid storm dx, identified from discharge code of index hospitalization	0 = No thyroid storm dx documented<br>1 = Diabetic coma dx	ICD-9: 242.01, 242.11, 242.21, 242.31, 242.41, 242.81, 242.91<br><br>ICD-10: E05.01, E05.11, E05.21, E05.31, E05.41, E05.81, E05.91
HR_RHABDOMYOL	Rhabdomyolisis dx identified at index hospitalization	Rhabdomyolisis dx, identified from discharge code of index hospitalization	0 = No rhabdomyolisis dx documented<br>1 = Rhabdomyolisis dx	ICD-9: 728.88<br><br>ICD-10: M62.82

HR_MYOSITIS	Myositis dx identified at index hospitalization	Myositis dx, identified from discharge code of index hospitalization	0 = No myositis dx documented<br>1 = Myositis dx	ICD-9: 728<br><br>ICD-10: M60
HR_MYOPATHY	Critical illness myopathy dx identified at index hospitalization	Critical illness myopathy dx, identified at index hospitalization	0 = No critical illness myopathy dx documented<br>1 = Critical illness myopathy dx	ICD-9: 359.81<br><br>ICD-10: G72.81

Variable Name	Variable Label	Variable Description	Value Labels	Notes
**VII. Pregnancy History and at Admission**				
PREG_GA_ADM	GA at hosp admission	Gestational age of fetus at hospital admission (days) ADM_DT minus PREG_LMP_DT	Completed weeks with up to 2 decimal places for the number of days (obtained by dividing the number of days by 7)	
PREG_TRI	Trimester at admission	Trimester at admission	Per ACOG:<br>1 = First trimester: day 14 through 13 weeks + 6 days (i.e., day 14 to day 97)<br>2 = Second trimester: 14 weeks + 0 days through 27 weeks + 6 days (i.e., day 98 to day 195)<br>3 = Third trimester: 28 weeks + 0 days through the end of pregnancy (i.e., day 196 to end of pregnancy)	
PC_DI	Pregnancy complication identified at index hospitalization dichotomy	Any pregnancy complication identified from index hospitalization discharge codes dichotomy	0 = No pregnancy complication documented at index hospitalization<br>1 = One or more pregnancy complication identified	See list of variables below for ICD codes
PC_HEMOR	Hemorrhage in early pregnancy	Identified by diagnostic codes during discharge from index hospitalization	0 = No dx documented<br>1 = Dx during index hospitalization as documented by diagnostic code	ICD-9: 640.00, 640.01, 640.03, 640.80, 640.81, 640.83, 640.90, 640.91, 640.93<br><br>ICD-10: O20.0, O20.8, O20.9

PC_BLEED	Bleeding in pregnancy	Identified by diagnostic codes during discharge from index hospitalization	0 = No dx documented<br>1 = Dx during index hospitalization as documented by diagnostic code	ICD-9: 641.00, 641.01, 641.03, 641.10, 641.11, 641.13, 461.20, 641.21, 641.23, 641.30, 641.31, 641.33, 641.80, 641.81, 641.83, 641.90, 641.91, 641.93, 649.3, 649.5<br><br>ICD-10: O26.851-O26.853 (no equiv ICD-10-CA code), O26.859 (no equiv ICD-10-CA code), O44 (ICD-10-CA: O44101, O44103, O44109), O45<br>ICD-10-AM: Would be recorded as O99.01 followed by D50-D64 (inclusive)
PC_HYPRTNSN	Hypertension complicating pregnancy	Identified by diagnostic codes during discharge from index hospitalization	0 = No dx documented<br>1 = Dx during index hospitalization as documented by diagnostic code	ICD-9: 642.00-642.04, 642.10-642.14, 642.20-642.24, 642.30-642.34, 642.40-642.44, 642.50-642.54, 642.60-642.64, 642.70-642.74, 642.90-642.94<br>ICD-10: O10.011-O10.013, O10.019, O10.02, O10.911-O10.913, O10.919, O10.92<br>ICD-10-CA: O10001-O10004, O10009, O10401-O10404, O10409, O10901-O10904, O10909
PC_VOMIT	Excessive vomiting during pregnancy	Identified by diagnostic codes during discharge from index hospitalization	0 = No dx documented<br>1 = Dx during index hospitalization as documented by diagnostic code	ICD-9: 643.00, 643.01, 643.03, 643.10, 643.11, 643.13, 643.20, 643.21, 643.23, 643.80, 643.81, 643.83, 643.90, 643.91, 643.93<br>ICD-10: O21.0-O21.2, O21.8, O21.9
PC_RENAL	Renal disease during pregnancy	Identified by diagnostic codes during discharge from index hospitalization	0 = No dx documented<br>1 = Dx during index hospitalization as documented by diagnostic code	ICD-9: 646.2<br>ICD-10: O26.83, O26.831-O26.839<br>ICD-10-CA: O10201-O10209, O10301-O10304
PC_INFCT	Infectious and parasitic diseases complicating pregnancy	Identified by diagnostic codes during discharge from index hospitalization	0 = No dx documented<br>1 = Dx during index hospitalization as documented by diagnostic code	ICD-9: 647.00-647.04, 647.10-647.14, 647.20-647.24, 647.30-647.34, 647.40-647.44, 647.50-647.54 647.60-647.64, 647.80-647.84, 647.90-647.94<br>ICD-10: A70, A82, A83, A84, A85, A86, A90, A91, A92, A95, A96, A98, B25, B27, B97.11 (no equiv ICD-10-CA code), O85, O98.011, O98.012, O98.013, O98.019 (ICD-10-CA: O98001-O98004, O98009), R07.81<br>ICD-10-AM: O98.8, O98.5O99.4;<br>A82, A90, A91, B97.11, and R07.81 do not exist in Australia
PC_DIABT	Diabetes during pregnancy	Identified by diagnostic codes during discharge from index hospitalization	0 = No dx documented<br>1 = Dx during index hospitalization as documented by diagnostic code	ICD-9: 648.00-648.04, 648.80-648.84<br>ICD-10: O24.0, O24.1, O24.3, O24.4, O24.8, O24.9 (inclusive) – BUT NOTE THAT THIS INCLUDES BOTH PREEXISTING AND GESTATIONAL DIABETES

PC_THYRD	Thyroid dysfunction in pregnancy	Identified by diagnostic codes during discharge from index hospitalization	0 = No dx documented<br>1 = Dx during index hospitalization as documented by diagnostic code	ICD-9: 648.10-648.14<br>ICD-10: O90.5
PC_DRG_DEPEND	Drug dependence during pregnancy	Identified by diagnostic codes during discharge from index hospitalization	0 = No dx documented<br>1 = Dx during index hospitalization as documented by diagnostic code	ICD-9: 648.30-648.34<br>ICD-10: O99.32 (no equiv ICD-10-CA code)
PC_CVD	Congenital cardiovascular disorders complicating pregnancy	Identified by diagnostic codes during discharge from index hospitalization	0 = No dx documented<br>1 = Dx during index hospitalization as documented by diagnostic code	ICD-9: 648.50-648.54<br>ICD-10: O99.4<br>ICD-10-CA: O99401-O99404, O99409
PC_SMOKE	Maternal smoking at index admission	Identified by diagnostic codes during discharge from index hospitalization	0 = No dx documented<br>1 = Dx during index hospitalization as documented by diagnostic code<br>99 = Code does not exist in country's coding system	ICD-9: 649.0<br>ICD-10: O99.33 (no equiv ICD-10-CA or ICD-10-AM code)
PC_EPILEPSY	Epilepsy complicating pregnancy	Identified by diagnostic codes during discharge from index hospitalization	0 = No dx documented<br>1 = Dx during index hospitalization as documented by diagnostic code<br>99 = Code does not exist in country's coding system	ICD-9: 649.4<br>ICD-10: O99.35 (no equiv ICD-10-CA code)
PC_MALPOSTN	Malposition of fetus	Identified by diagnostic codes during discharge from index hospitalization	0 = No dx documented<br>1 = Dx during index hospitalization as documented by diagnostic code	ICD-9: 652<br>ICD-10: O32

PC_CEPHAL_DISP	Cephalopelvic disproportion	Identified by diagnostic codes during discharge from index hospitalization	0 = No dx documented<br>1 = Dx during index hospitalization as documented by diagnostic code	ICD-9: 653<br>ICD-10: O33
PC_ORG_ABNORM	Abnormalities of organs and soft tissues of pelvis	Identified by diagnostic codes during discharge from index hospitalization	0 = No dx documented<br>1 = Dx during index hospitalization as documented by diagnostic code	ICD-9: 654.00-654.04,654.10-654.14,654.20-654.24,654.30-654.34,654.40-654.44,654.50-654.54,654.60-654.64,654.70-654.74,654.80-654.84,654.90-654.94<br>ICD-10: O34
PC_FETUS_DRG	Suspected damage to fetus due to maternal drug use	Identified by diagnostic codes during discharge from index hospitalization	0 = No dx documented<br>1 = Dx during index hospitalization as documented by diagnostic code	ICD-9: 655.53<br>ICD-10: O35.5<br>ICD-10-CA: O35501, O35503, O35509
PC_FETAL_GRWTH	Fetal growth restriction	Identified by diagnostic codes during discharge from index hospitalization	0 = No dx documented<br>1 = Dx during index hospitalization as documented by diagnostic code	ICD-9: 656.53<br>ICD-10: O36.5
PC_PRETERM	Early or threatened labor	Identified by diagnostic codes during discharge from index hospitalization	0 = No dx documented<br>1 = Dx during index hospitalization as documented by diagnostic code	ICD-9: 644.0, 644.2, 649.7, 654.0, 654.1, 654.3, 654.4, 654.5, 654.6, 657, 658.0, 658.4<br>ICD-10: O26, O34, O40-O42, O60-O69

PC_OTHER	Other pregnancy complication	Identified by diagnostic codes during discharge from index hospitalization	0 = No dx documented<br>1 = Dx during index hospitalization as documented by diagnostic code	ICD-9: 052-055, 060-066, 070, 071, 073, 099.1, 099.3, 262, 263, 280.1, 280.8, 280.9, 291-293, 295-299, 301, 303, 305, 307.1, 307.5, 308, 309, 443.2, 449, 459.0, 459.1, 459.2, 459.3, 645, 646.0, 646.3, 646.6, 646.7, 648.6, 649.3, 651, 653.6, 656.0, 656.3, 656.4, 656.6, 656.7, 656.8, 656.9<br><br>ICD-10: A55, A60, B01.0, B01.11, B01.12, B01.2, B01.81, B01.89, B02.39, B15-B19, D59.6, F04-F06, F10-F16, F18-F19, F20, F29, F30-F34, F43, F48.8, F48.9, F53, F60, F68.8, F69, F84.0, F84.3, F84.8, F84.9, I81, I82, I87, I95, M02, N96, O10-O16, O20.8, O20.9, O26.20-23, O30, O31, O43, O45, O48.1, O98.02, O98.03, O98.111-119, O98.211-219, O98.22, O98.23, O98.711, O98.712, O98.713, O98.719, R82.3<br><br>ICD-10-CA alternatives for those ICD-10 that do not exist: B0111, B0112, B0181, B0189, B0239, O2621, O2622, O2623, O481, O9802, O9803, O98111, O98112, O98113, O98114, O98115, O98116, O98117, O98118, O98119, O98211, O98212, O98213, O98214, O98215, O98216, O98217, O98218, O98219, O9822, O9823, O98711, O98712, O98713, O98719
MAT_GRAVIDITY	Gravidity – total number of previous pregnancies regardless of the outcome. Note: a multiple pregnancy is considered as one pregnancy.		0 – 10<br>99 = missing or unknown	
MAT_PARITY	Parity – total number of previous pregnancies that reached a viable gestational age (includes live births and stillbirths). Note: a multiple pregnancy is considered as one pregnancy.		0 – 10<br>99 = missing or unknown	

MAT_PRIMIPAR	Indicates whether mother is primiparous (includes live births and stillbirths). Note: a multiple pregnancy is considered as one pregnancy.		0 = Primiparous (MAT_PARITY_NUM=0)<br>1 = Not primiparous (1≤MAT_PARITY_NUM<99)	
MAT_PARITY_NUM	Analytic variable to categorize MAT_PARITY		0 = MAT_PARITY = 0<br>1 = MAT_PARITY = 1<br>2 = MAT_PARITY = 2-10<br>99 = MAT_PARITY = 99	
PREG_MULT	Pregnancy singleton or multiple		1 = One fetus<br>2 = More than one fetus<br>99 = Unknown	
PREG_GA_AV	GA at first antenatal visit	Gestational age of fetus at first antenatal visit	Completed weeks with up to 2 decimal places for the number of days (obtained by dividing the number of days by 7)<br>99 = Unknown	
PREG_AV_ADM	Antenatal visits prior to hospitalization	Number of antenatal visits prior to hospital admission	0 – XX<br>99 = Unknown	

Variable Name	Variable Label	Variable Description	Value Labels	Notes
**VIII. Hospital Event Description**				
HOSP_ADM_DATE	Index hosp admission date	Index hospital admission date		
HOSP_DC_DATE	Index hosp discharge date	Index hospital discharge date		
HOSP_ADM_TIME	Index hosp admission time	Index hospital admission time – in 24:00 format	99 = Unknown or could not be determined	
HOSP_DC_TIME	Index hosp discharge time	Index hosp discharge time – in 24:00 format	99 = Unknown or could not be determined	
HOSP_24	Index hosp admission >24 hours	Index hospital admission lasted greater than 23 hours		
HOSP_YR	Index hospital admission year	Index year of hospital admission	2010 to 2016	
HOSP_WK	Index hospital admission week	Index week of hospital admission (using MMWR week windows)	0 – 53	Will use date windows for MMWR weeks specific to each year. Week #1 of an MMWR year is the first week of the year that has at least four days in the calendar year. https://ibis.health.state.nm.us/resource/MMWRWeekCalendar.html
HOSP_DAYS	Index hospitalization days	Index total days hospitalized (Discharge minus admission date)	0 – 100+	In cases of readmission within 14 days, only days from the first hospitalization are included.
HOSP_HOURS	Index hospitalization hours	Index total hours hospitalized (from admission hour to discharge hour)	9999 = unknown or unavailable	Will be unknown for sites unable to use time variables
ICU_ANY	ICU admission during index hospitalization	Dichotomous indicator of any ICU admission during hospitalization	0 = No ICU admission with this hospitalization<br>1 = ICU admission during hospitalization<br>99 = Unknown or no way to determine	

ICU_ADM_DT	ICU admission date	ICU admission date	99 = Unknown or could not be determined	If woman was discharged and then re-admitted to the ICU within the same hospitalization, use first admission date/time
ICU_ADM_TIME	ICU admission time	ICU admission time – in 24:00 format	99 = Unknown or could not be determined	If woman was discharged and then re-admitted to the ICU within the same hospitalization, use first admission date/time
ICU_DISCH_DT	ICU discharge date	ICU discharge date	99 = Unknown or could not be determined	If woman was discharged and then re-admitted to the ICU within the same hospitalization, use last discharge date/time
ICU_DISCH_TIME	ICU discharge time	ICU time – in 24:00 format	99 = Unknown or could not be determined	If woman was discharged and then re-admitted to the ICU within the same hospitalization, use last discharge date/time
ICU_DAYS	ICU days	Total days in ICU (Discharge minus admission date)		
ICU_HOURS	ICU hours	Total hours in ICU (from admission hour to discharge hour)	99 = Unknown or could not be determined	
HOSP_DC_CODE1-15	Hospital discharge code variables			
HOSP_DC_PRIME	Primary discharge code			
HOSP_DCSTATUS	Index hosp discharge status	Index hosp discharge status	1 = Discharged home<br>2 = Discharged to long term care facility<br>3 = Transferred to another hospital or rehabilitation facility<br>4 = Died while hospitalized<br>5 = Other	Describe where patient discharged to or whether patient died while hospitalized
HOSP_VENT	Ventilation received during index hospitalization	Ventilation received during index hospitalization	0 = No ventilation<br>1 = Ventilation received<br>99 = Unknown	
HOSP_ECMO	Requirement for ECMO during index hospitalization	Requirement for ECMO during index hospitalization	0 = No ECMO<br>1 = ECMO received<br>99 = Unknown	ICD-10 procedure code: 5A15223<br><br>ICD-9 procedure code: 39.65

HOSP_ANTIVIRL	Received influenza antiviral medication during index hospitalization	Received influenza antiviral medication during index hospitalization	0 = No antiviral received<br>1 = Antiviral received<br>99 = Unknown	
HOSP_ANTIVIRL_DAYS	Days between admission for index hospitalization and first antiviral dose	Days between admission for index hospitalization and first antiviral dose	99 = No antiviral received or unknown	
HOSP_DELIV	Indicates whether the index hospitalization was also the delivery hospitalization	Indicates whether the index hospitalization was also the delivery hospitalization	0 = Pregnancy end date does not fall between hospitalization start and end date<br>1 = Pregnancy end date falls between hospitalization start and end date<br>99 = Unknown	
HOSP_GEST_GP	Indicates when index hospital admission occurred during pregnancy	Indicates when index hospital admission occurred during pregnancy	1 = Admission at <14 weeks gestation<br>2 = Admission at 14-27 weeks gestation<br>3 = Admission at 28-36 weeks gestation<br>4 = Admission at ≥37 weeks gestation	Variable needed to inform exclusions for preterm analysis
HOSP_READMIT	Readmission for qualifying ARI/F event within 14 days of discharge date of index hospitalization	Readmission for qualifying ARI/F event within 14 days of discharge date of index hospitalization	0 = No qualifying readmission within 14 days<br>1 = Qualifying readmission within 14 days<br>99 = Unknown	Intended to count the number of instances where clinical testing may have been done in two temporally close hospitalizations. Both admissions must be within 14 days of one another and have a qualifying ARI/F discharge diagnosis. Only discharge diagnoses from the first hospitalization are included in the analysis.

Variable Name	Variable Label	Variable Description	Value Labels	Notes
**IX. Illness Onset**				
ILL_ONSET_CONF	ARI/F illness onset confirmed	ARI/F illness onset date confirmation by category	1 = onset listed and within 10 days of admission<br>2 = onset listed, but >10 days of admission<br>99 = no onset date available	Admission date is day zero<br><br>Illness refers to the ARI/febrile illness that resulted in the index hospitalization.
ILL_ONSET_DT	Illness onset date		99 = Unknown	From protocol: two days prior to admission date if unknown
ILL_ONSET_DAYS	Days since illness onset	Days from admission to illness onset (HOSP_ADM_DT minus ILL_ONSET_DT)	99 - Unknown	Admission date is day zero

Variable Name	Variable Label	Variable Description	Value Labels	Notes
**X. Virus Testing**				
FLU_METHOD_ANY	Flu tested by any assay	Flu tested by any assay, including RT-PCR, RIDT, DFA, culture, or other; during hospitalization or 3 days prior to admission	0 = Not tested with any assay<br>1 = Tested with any assay, including RT-PCR, RIDT, DFA, culture, serology, or other	
FLU_TEST_PRIOR	Flu testing occurred prior to hospitalization, within 3 day prior	Flu testing occurred prior to hospitalization, within 3 day prior	0 = Testing occurred during hospitalization<br>1 = Testing occurred in 3 days prior to admission	
FLU_METHOD_SUM	Flu tested by PCR or Other	Flu tested by PCR or other lab	0 = Not tested for flu<br>1 = Tested by PCR for flu<br>2 = Tested by non-PCR lab for flu	If FLU_METHOD_ANY EQ 1 but FLU_RTPCR EQ 99 then FLU_METHOD_SUM EQ 2
FLU_CONF	Confirmed flu by any method	Confirmed flu by any method	0 = Flu negative<br>1 = Flu positive<br>99 = Excluded – No PCR result	1.
FLU_RTPCR	Flu outcome by RT-PCR universal A and B	Flu outcome for primary analysis relying on RT-PCR only - earliest test result during hospitalization or 3 days prior to admission is default	0 = PCR confirmed influenza negative<br>1 = PCR confirmed influenza A or B positive<br>99 = Excluded - No PCR result	
FLU_RTPCR_A	Flu A by RT-PCR	Confirmed influenza A by RT-PCR	0 = Not influenza A<br>1 = A influenza confirmed by RT-PCR	
FLU_RTPCR_AH3N2	Flu A H3N2 subtype by RT-PCR	Confirmed A H3N2 subtype by RT-PCR	0 = Not A H3N2<br>1 = A H3N2 subtype confirmed by RT-PCR	

FLU_RTPCR_AH1PDM	Flu A H1N1 PDM subtype by RT-PCR	Confirmed A H1N1 pdm09 subtype by RT-PCR	0 = Not A H1N1 pdm09<br>1 = A H1N1 pdm09 subtype confirmed by RT-PCR	
FLU_RTPCR_B	Flu B by RT-PCR	Confirmed influenza B typed by RT-PCR	0 = Not influenza B<br>1 = Influenza B confirmed by RT-PCR	
SPEC_DT	Specimen collection date	Specimen collection date - earliest test result during hospitalization or 3 days prior to admission is default		
SPEC_HR	Specimen collection hour	Specimen collection hour - earliest test result during hospitalization or 3 days prior to admission is default		
SPEC_24ADM	Specimen collection within 24 hours of admission	Specimen collection within 24 hours of admission	0 = specimen collected >24 hours after admission<br>1 = specimen confirmed collected within 24 hours of admission<br>9 = unknown or unavailable	
FLU_METHOD_RIDT	Flu tested by RIDTs	Flu tested by rapid diagnostic tests RIDTs - earliest test result during hospitalization or 3 days prior to admission is default	0 = RIDT flu negative<br>1 = RIDT flu positive<br>99 = No RIDT testing	
FLU_METHOD_DFA	Flu tested by DFA/IFA	Flu tested by DFA/IFA – earliest test result during hospitalization or 3 days prior to admission is default	0 = DFA/IFA flu negative<br>1 = DFA/IFA flu positive<br>99 = No DFA/IFA testing	

FLU_METHOD_CULTURE	Flu tested by viral culture	Flu tested by viral culture – earliest test result during hospitalization or 3 days prior to admission is default	0 = Viral culture flu negative<br>1 = Viral culture flu positive<br>99 = No viral culture testing	
FLU_METHOD_OTHER	Flu tested by other assays	Flu tested by other non RT-PCR assays during hospitalization or 3 days prior to admission	0 = OTHER flu negative<br>1 = OTHER flu A positive<br>2 = OTHER flu B positive<br>3 = OTHER flu positive, unspecified<br>99 = No OTHER testing	
RSV_TESTED	RSV tested by any assay	RSV tested by an assay, including RT-PCR, DFA, culture or other during hospitalization or 3 days prior to admission	0=Not tested with any assay<br>1 = Tested with an assay, including RT-PCR, DFA, culture, serology, or other	
RSV_TEST_METHOD	Testing method used to detect RSV	Laboratory testing method used to detect RSV - earliest test result during hospitalization or 3 days prior to admission is default	1 = RT-PCR<br>2 = DFA<br>3 = Culture<br>4 = Serology<br>5 = Other	
RSV_RTPCR_RESULT	RSV outcome by RT-PCR	RSV detection made by RT-PCR only – earliest test result during hospitalization or 3 days prior to admission is default	0 = PCR confirmed RSV negative<br>1 = PCR confirmed RSV positive<br>99 = Excluded – No PCR	
RSV_RESULT	RSV result	RSV result - earliest test result during hospitalization or 3 days prior to admission is default	0 = RSV negative<br>1 = RSV positive<br>99 = Not tested	

RSV_POS_AB	RSV type A or B	RSV type A or B viruses – earliest test result during hospitalization or 3 days prior to admission is default	0 = RSV negative<br>1 = RSV A virus<br>2 = RSV B virus<br>99 = Not tested or unknown	
hMPV_RESULT	hMPV result	hMPV result - earliest test result during hospitalization or 3 days prior to admission is default	0 = hMPV negative<br>1 = hMPV positive<br>99 = Not tested	
PARA_RESULT	Parainfluenza result	Parainfluenza result – earliest test result during hospitalization or 3 days prior to admission is default	0 = Para negative<br>1 = Para positive<br>99 = Not tested	
ADENO_RESULT	Adenovirus result	Adenovirus result – earliest test result during hospitalization or 3 days prior to admission is default	0 = Adeno negative<br>1 = Adeno positive<br>99 = Not tested	
CORONA_RESULT	Coronavirus result	Coronavirus result – earliest test result during hospitalization or 3 days prior to admission is default	0 = Corona negative<br>1 = Corona positive<br>99 = Not tested	
RHINO_RESULT	Rhinovirus result	Rhinovirus result – earliest test result during hospitalization or 3 days prior to admission is default	0 = Rhino negative<br>1 = Rhino positive<br>99 = Not tested	

ENTERO_RESULT	Enterovirus result	Enterovirus result – earliest test result during hospitalization or 3 days prior to admission is default	0 = Entero negative<br>1 = Entero positive<br>99 = Not tested	
OTHER_VIRUS_RESULT	Other non influenza virus result	Other non influenza virus result - earliest test result during hospitalization or 3 days prior to admission is default	0 = Other non influenza virus negative<br>1 = Other non influenza virus positive<br>99 = Not tested	

Variable Name	Variable Label	Variable Description	Value Labels	Notes
**XI. Influenza Vaccination**				
VX_SEASON_DT	Current season vx date	Current season influenza vaccination date		
VX_SEASON_DAYS	Days from vx date to hospital admission	Days from influenza vaccination date to hospital admission	0 - 364<br>999 = not available	
VX_SEASON_ADM	Current season vx delivered	Current season influenza vaccination delivered	0 = Not vaccinated<br>1 = Vaccinated 0-14 days prior to admission<br>2 = Vaccinated >14 days from admission	Those known to be vaccinated 0-14 days prior to admission will be excluded from the analytic sample as immunization status indeterminate
VX_SEASON_FINAL	Current season vx or not	Current season influenza vaccinated >14 days prior to admission	0 = Unvaccinated<br>1 = Vaccinated	This is the final analytic vaccination variable. Those known to be vaccinated 0-14 days from hospitalization are excluded from analysis. Thus, a patient is vaccinated with any accepted vaccination documentation and known to be vaccinated >14 days or missing vaccination date and thus assumed to be >14 days

VX_SEASON_PRIOR	Vaccination for both current and prior season	Vaccination for both current and prior season	0 = Unvaccinated both current and prior season<br>1 = Vaccinated in prior season only<br>2 = Vaccinated in current season only<br>3 = Vaccinated in both current and prior season<br>99 = Unknown or not in prior season data	Sites will code as 99 women who would not be expected to have prior season vaccination records because they were not health plan members or did not live in study area when vaccine became widely available (such as October 1st in NH sites).

Variable Name	Variable Label	Variable Description	Value Labels	Notes
**XII. Birth Outcomes**				
OUT_ADVERSE	Adverse birth outcome	An adverse birth outcome	1 = Adverse birth outcome<br>0 = No adverse birth outcome	Coded 1 if:<br>PREG_TRI = 3 AND (OUT_PRETERM=1 OR OUT_STILLBIRTH=1)
OUT_PRETERM	Preterm birth	A live birth with an estimated gestation <37 weeks	1 = Preterm infant<br>0 = Term infant<br>99 = Ineligible (e.g., stillbirth)	
OUT_SGA	Small for gestational age birth	A live birth where the infant is born in the lowest 10th percentile for gender and gestational age	1 = Small for gestational age birth<br>0 = No SGA birth	Sites will use appropriate local or national benchmark values for birthweight percentiles by gestational age and gender to determine the lowest 10th percentile.
OUT_LBW	Low birthweight	A live birth where the infant is born <2500 grams	1 = Low birthweight<br>0 = Not low birthweight	
OUT_STILLBIRTH	Stillborn infant	Infant born >=20 weeks gestation with no sign of life	1 = Stillborn infant<br>0 = No stillbirth	

OUT_FLU_HOSP_STATUS	Exposure status category	Category of exposure for analysis of birth outcomes	0 = No record of hospital admission for ARI during pregnancy*<br>1 = Admissions for ARI during pregnancy and RT-PCR NEGATIVE for influenza (FLU_RTPCR=0)<br>2 = Admitted for ARI during pregnancy and RT-PCR POSITIVE for influenza (FLU_RTPCR=1)	*For non-hospitalized women, this includes women who have a record of pregnancy overlapping with the pre-defined, site-specific influenza season, but had no record of admission to hospital with ARI. NOTE: some sites have information available on non-admitted influenza cases. If possible, exclude these from the non-hospitalized group (0).
OUT_QTR_CONCEPT	Quarter of conception	Estimated quarter of calender year of pregnancy start	1 = Jan-Mar<br>2=Apr-Jun<br>3=Jul-Sep<br>4=Oct-Dec<br>99=Unknown	Variable will be used to time match exposed and unexposed pregnancies
OUT_VP_HR_DI	High risk medical conditions identified at any point in the year prior to pregnancy start date dichotomy	High risk medical condition (chronic condition, not pregnancy complication) identified from medical records in the one year prior to pregnancy start date	0 = No high risk medical condition (not pregnancy complication) documented<br>1 = One or more high risk medical condition(s) documented	This is the same variable as the Vaccination Patterns paper, but with obesity as a HR condition (MAT_BMI_PRIOI_OBESE)

Variable Name	Variable Label	Variable Description	Value Labels	Notes
**XIII. Vaccination Patterns**				
VP_PID	Participant ID	Participant ID - uniquely associated with each individual like medic rec # or unique ID for your site	Site specific variable	Same woman can appear more than once in the dataset, but only once per influenza season.

VP_SEASON_YEAR	Influenza season in which pregnancy occurs	Site-specific influenza season.	"2010/2011"<br>"2011/2012"<br>"2012/2013"<br>"2013/2014"<br>"2014/2015"<br>"2015/2016"	This variable defines the window for population capture. Pregnancy overlapping specified flu season is captured.
VP_MAT_AGE_35	Maternal age as of pregnancy start date, LT or GE 35 years	Maternal age as of pregnancy start date, LT or GE 35 years	1 = < 35 years<br>2 = >= 35 years<br>99 = unknown	
VP_HR_DI	High risk medical conditions identified at any point in the year prior to pregnancy start date dichotomy	High risk medical condition (chronic condition, not pregnancy complication) identified from medical records in the one year prior to pregnancy start date	0 = No high risk medical condition (not pregnancy complication) documented<br>1 = One or more high risk medical condition(s) documented	Use code list from HR_DI
VP_PC_DI	Pregnancy complication identified at any point in the pregnancy (start date through end date) dichotomy	Any pregnancy complication identified from medical records identified at any point in the pregnancy (start date through end date)	0 = No pregnancy complication documented<br>1 = One or more pregnancy complications documented	Use code list from PC_DI
VP_VX_DI	Current season vx or not	Current season influenza vaccinated, anytime within the vaccine availability period (8/1 through 4/30 of influenza season OR end of flu season, whichever is later)	0 = Unvaccinated<br>1 = Vaccinated	Vaccination Status for the flu season, but allowing vaccines from Aug 1 – ENDDT, where ENDDT is the MAXIMUM of 4/30 or the end of the flu season.

VP_VX_TIMING	Current season vx timing, relative to pregnancy	Current season vx timing, relative to pregnancy	0 = Unvaccinated<br>1 = Prior to pregnancy start date<br>2 = 1st trimester<br>3 = 2nd trimester<br>4 = 3rd trimester<br>5 = After pregnancy end date	
VP_SEASON_FLU_PERIOD	Current season vx timing, relative to flu circulation	Current season vx timing, relative to flu circulation	0 = Unvaccinated<br>1 = Vaccinated prior to early season<br>2 = Vaccinated during early season with sustained local circulation at low levels<br>3 = Vaccinated during high and peak periods of circulation<br>4 = Vaccinated during late season with sustained local circulation at low levels<br>5 = Vaccinated after late season	Early, peak, and late periods defined by site for SEASON_FLU_PERIOD variable

Variable Name	Variable Label	Variable Description	Value Labels	Notes
**XIV. Analysis Specification**				
ANALYSIS_FLU_VE	Record included in VE analysis		0 – Record not included in VE analysis<br>1 – Record included in VE analysis	Record is included in VE analysis if all of the following criteria are met:<br>a. Woman age 18-50 at time of inpatient admission.<br>b. Pregnant at time of admission.<br>c. Date of admission during flu season 2010/11 thru 2015/16<br>d. Eventual pregnancy outcome of LB or SB at >= 20 weeks gestational age.<br>e. Discharge diagnosis of ARI/Febrile illness<br>f. Flu PCR tested during hospitalization or in 3 days prior to admission.<br>g. Vaccination status documented (excludes those with vaccination 0-14 days prior to admission; if VX_SEASON_ADM = 1).
ANALYSIS_RSV	Record included in RSV analysis		0 – Record not included in RSV analysis<br>1 – Record included in RSV analysis	Record is included in RSV analysis if:<br>a. Woman was pregnant during RSV season<br>b. Had an eventual pregnancy outcome of LB or SB at >=20 weeks gestational age
ANALYSIS_VAC_NOTVAC	Record included in vac/not vac analysis		0 – Record not included in vac/not vac analysis<br>1 – Record included in vac/not vac analysis	Detailed specifications needed for the vac/not vac analysis.

APPENDIX: Primary Code Sets

Description	ICD-10 Code	ICD-10-AM Code	ICD-10-CA Code	Notes
**Acute Respiratory or Febrile Illness (ARI/F) Diagnostic Codes**				
Viral infection of unspecified site	B34	B34	B34	
Adenovirus infection, unspecified	B34.0	B34.0	B34.0	
Enterovirus infection, unspecified	B34.1	B34.1	B34.1	
Coronavirus infection, unspecified	B34.2	B34.2	B34.2	
Parvovirus infection, unspecified	B34.3	B34.3	B34.3	
Papovavirus infection, unspecified	B34.4	B34.4	B34.4	
Other viral infection, unspecified	B34.8	B34.8	B34.8	
Viral infection, unspecified	B34.9	B34.9	B34.9	
Respiratory syncytial virus as the cause of diseases classified elsewhere	B97.4	B97.4	B97.4	
Other viral agents as the cause of diseases classified elsewhere	B97.8	B97.8	B97.8	
Acute nasopharyngitis	J00	J00	J00	
Acute sinusitis	J01	J01	J01	
Acute maxillary sinusitis, unspecified	J01.00	J01.0	J01.00	
Acute recurrent maxillary sinusitis	J01.01	J01.0	J01.01	
Acute frontal sinusitis, unspecified	J01.10	J01.1	J01.10	
Acute recurrent frontal sinusitis	J01.11	J01.1	J01.11	
Acute ethmoidal sinusitis, unspecified	J01.20	J01.2	J01.20	
Acute recurrent ethmoidal sinusitis	J01.21	J01.2	J01.21	
Acute sphenoidal sinusitis, unspecified	J01.30	J01.3	J01.30	
Acute recurrent sphenoidal sinusitis	J01.31	J01.3	J01.31	
Acute pansinusitis, unspecified	J01.40	J01.4	J01.40	
Acute recurrent pansinusitis	J01.41	J01.4	J01.41	
Other acute sinusitis	J01.80	J01.8	J01.80	
Other acute recurrent sinusitis	J01.81	J01.8	J01.81	
Acute sinusitis, unspecified	J01.90	J01.9	J01.90	
Acute recurrent sinusitis, unspecified	J01.91	J01.9	J01.91	
Acute pharyngitis	J02	J02	J02	
Streptococcal pharyngitis	J02.0	J02.0	J02.0	

Acute pharyngitis due to other specified organisms	J02.8	J02.8	J02.8	
Acute pharyngitis, unspecified	J02.9	J02.9	J02.9	
Acute tonsillitis	J03	J03	J03	
Acute streptococcal tonsillitis, unspecified	J03.00	J03.0	J03.00	
Acute recurrent streptococcal tonsillitis	J03.01	J03.8	J03.01	
Acute tonsillitis due to other specified organisms	J03.80	J03.8	J03.80	
Acute recurrent tonsillitis due to other specified organisms	J03.81	J03.9	J03.81	
Acute tonsillitis, unspecified	J03.90	J03.8	J03.90	
Acute recurrent tonsillitis, unspecified	J03.91	J03.8	J03.91	
Acute laryngitis and tracheitis	J04	J04	J04	
Acute laryngitis	J04.0	J04.0	J04.0	
Acute tracheitis without obstruction	J04.10	J04.1	J04.10	
Acute tracheitis with obstruction	J04.11	J04.1	J04.11	
Acute laryngotracheitis	J04.2	J04.2	J04.2	
Supraglottitis, unspecified, without obstruction	J04.30	*Code does not exist*	J04.30	
Supraglottitis, unspecified, with obstruction	J04.31	*Code does not exist*	J04.31	
Acute upper respiratory infections of multiple and unspecified sites	J06	J06	J06	
Acute laryngopharyngitis	J06.0	J06.0	J06.0	
Other acute upper respiratory infections of multiple sites	J06.8	J06.8	J06.8	
Acute upper respiratory infection, unspecified	J06.9	J06.9	J06.9	
Influenza due to certain identified influenza viruses	J09	J09	J09	
Influenza due to identified novel influenza A virus	J09.X	J09	J09.X	
Influenza due to identified novel influenza A virus with pneumonia	J09.X1	J09	J09.X1	

Influenza due to identified novel influenza A virus with other respiratory manifestations	J09.X2	J09	J09.X2	
Influenza due to identified novel influenza A virus with gastrointestinal manifestations	J09.X3	J09	J09.X3	
Influenza due to identified novel influenza A virus with other manifestations	J09.X9	J09	J09.X9	
Influenza due to other identified influenza virus	J10	J10	J10	
Influenza due to other identified influenza virus with pneumonia	J10.0	J10.0	J10.0	
Influenza due to other identified influenza virus with unspecified type of pneumonia	J10.00	J10.0	J10.00	
Influenza due to other identified influenza virus with the same other identified influenza virus pneumonia	J10.01	J10.0	J10.01	
Influenza due to other identified influenza virus with other specified pneumonia	J10.08	J10.0	J10.08	
Influenza due to other identified influenza virus with other respiratory manifestations	J10.1	J10.1	J10.1	
Influenza due to other identified influenza virus with gastrointestinal manifestations	J10.2	Code does not exist	J10.2	
Influenza due to other identified influenza virus with other manifestations	J10.8	J10.8	J10.8	
Influenza due to other identified influenza virus with encephalopathy	J10.81	J10.8	J10.81	
Influenza due to other identified influenza virus with myocarditis	J10.82	J10.8	J10.82	
Influenza due to other identified influenza virus with otitis media	J10.83	J10.8	J10.83	

Influenza due to other identified influenza virus with other manifestations	J10.89	J10.8	J10.89	
Influenza due to unidentified influenza virus	J11	J11	J11	
Influenza due to unidentified influenza virus with pneumonia	J11.0	J11.0	J11.0	
Influenza due to unidentified influenza virus with unspecified type of pneumonia	J11.00	J11.0	J11.00	
Influenza due to unidentified influenza virus with specified pneumonia	J11.08	J11.0	J11.08	
Influenza due to unidentified influenza virus with other respiratory manifestations	J11.1	J11.1	J11.1	
Influenza due to unidentified influenza virus with gastrointestinal manifestations	J11.2	Code does not exist	J11.2	
Influenza due to unidentified influenza virus with other manifestations	J11.8	J11.8	J11.8	
Influenza due to unidentified influenza virus with encephalopathy	J11.81	J11.8	J11.81	
Influenza due to unidentified influenza virus with myocarditis	J11.82	J11.8	J11.82	
Influenza due to unidentified influenza with otitis media	J11.83	J11.8	J11.83	
Influenza due to unidentified influenza virus with other manifestations	J11.89	J11.8	J11.89	
Viral pneumonia, not elsewhere classified	J12	J12	J12	
Adenoviral pneumonia	J12.0	J12.0	J12.0	
Respiratory syncytial virus pneumonia	J12.1	J12.1	J12.1	
Parainfluenza virus pneumonia	J12.2	J12.2	J12.2	
Human metapneumovirus pneumonia	J12.3	J12.3	J12.3	
Other viral pneumonia	J12.8	J12.8	J12.8	
Pneumonia due to SARS-associated coronavirus	J12.81	J12.8	J12.81	
Other viral pneumonia	J12.89	J12.8	J12.89	

Viral pneumonia, unspecified	J12.9	J12.9	J12.9	
Pneumonia due to Streptococcus pneumoniae	J13	J13	J13	
Pneumonia due to Hemophilus influenzae	J14	J14	J14	
Bacterial pneumonia, not elsewhere classified	J15	J15	J15	
Pneumonia due to Klebsiella pneumoniae	J15.0	J15.0	J15.0	
Pneumonia due to Pseudomonas	J15.1	J15.1	J15.1	
Pneumonia due to staphylococcus	J15.2	J15.2	J15.2	
Pneumonia due to staphylococcus, unspecified	J15.20	J15.2	J15.20	
Pneumonia due to Methicillin susceptible Staphylococcus aureus	J15.211	J15.2	J15.211	
Pneumonia due to Methicillin resistant Staphylococcus aureus	J15.212	J15.2	J15.212	
Pneumonia due to other staphylococcus	J15.29	J15.2	J15.29	
Pneumonia due to streptococcus, group B	J15.3	J15.3	J15.3	
Pneumonia due to other streptococci	J15.4	J15.4	J15.4	
Pneumonia due to Escherichia coli	J15.5	J15.5	J15.5	
Pneumonia due to other aerobic Gram-negative bacteria	J15.6	J15.6	J15.6	
Pneumonia due to Mycoplasma pneumoniae	J15.7	J15.7	J15.7	
Pneumonia due to other specified bacteria	J15.8	J15.8	J15.8	
Unspecified bacterial pneumonia	J15.9	J15.9	J15.9	
Pneumonia due to other infectious organisms, not elsewhere classified	J16	J16	J16	
Chlamydial pneumonia	J16.0	J16.0	J16.0	
Pneumonia due to other specified infectious organisms	J16.8	J16.8	J16.8	

Pneumonia in diseases classified elsewhere	J17	J17	J17	
Pneumonia, unspecified organism	J18	J18	J18	
Bronchopneumonia, unspecified organism	J18.0	J18.0	J18.0	
Lobar pneumonia, unspecified organism	J18.1	J18.1	J18.1	
Hypostatic pneumonia, unspecified organism	J18.2	J18.2	J18.2	
Other pneumonia, unspecified organism	J18.8	J18.8	J18.8	
Pneumonia, unspecified organism	J18.9	J18.9	J18.9	
Acute bronchitis	J20	J20	J20	
Acute bronchitis due to Mycoplasma pneumoniae	J20.0	J20.0	J20.0	
Acute bronchitis due to Hemophilus influenzae	J20.1	J20.1	J20.1	
Acute bronchitis due to streptococcus	J20.2	J20.2	J20.2	
Acute bronchitis due to coxsackievirus	J20.3	J20.3	J20.3	
Acute bronchitis due to parainfluenza virus	J20.4	J20.4	J20.4	
Acute bronchitis due to respiratory syncytial virus	J20.5	J20.5	J20.5	
Acute bronchitis due to rhinovirus	J20.6	J20.6	J20.6	
Acute bronchitis due to echovirus	J20.7	J20.7	J20.7	
Acute bronchitis due to other specified organisms	J20.8	J20.8	J20.8	
Acute bronchitis, unspecified	J20.9	J20.9	J20.9	
Acute bronchiolitis	J21	J21	J21	
Acute bronchiolitis due to respiratory syncytial virus	J21.0	J21.0	J21.0	
Acute bronchiolitis due to human metapneumovirus	J21.1	J21.1	J21.1	
Acute bronchiolitis due to other specified organisms	J21.8	J21.8	J21.8	

Acute bronchiolitis, unspecified	J21.9	J21.9	J21.9	
Unspecified acute lower respiratory infection	J22	J22	J22	
Other specified diseases of upper respiratory tract	J39.8	J39.8	J39.8	
Disease of upper respiratory tract, specified	J39.9	J39.9	J39.9	
Bronchitis, not specified as acute or chronic	J40	J40	J40	
Acute respiratory distress syndrome	J80	J80	J80	
Pulmonary edema	J81	J81	J81	
Acute pulmonary edema	J81.0	J81	J81.0	
Chronic pulmonary edema	J81.1	J81	J81.1	
Acute respiratory failure	J96.0	J96.0	J96.0	
Acute respiratory failure, unspecified whether with hypoxia or hypercapnia	J96.00	J96.00	J96.00	
Acute respiratory failure with hypoxia	J96.01	J96.01	J96.01	
Acute respiratory failure with hypercapnia	J96.02	Code does not exist	J96.02	
Respiratory failure, unspecified, unspecified whether with hypoxia or hypercapnia	J96.90	J96.90	J9690	
Respiratory failure, unspecified with hypoxia	J96.91	J96.91	J9691	
Respiratory failure, unspecified with hypercapnia	J96.92	Code does not exist	J9699	
Other diseases of bronchus, not elsewhere classified	J98.09	J98.0	J980	
Atelectasis	J98.11	J98.1	J9810	
Other pulmonary collapse	J98.19	J98.1	J98.19	
Other disorders of lung	J98.4	J98.4	J98.4	
Other specified respiratory disorders	J98.8	J98.8	J98.8	
Respiratory disorder, unspecified	J98.9	J98.9	J98.9	
Pyrexia during labor, not elsewhere classified	O75.2	O75.2	O75201, O75203, O75209	

Pyrexia of unknown origin following delivery	O86.4	O86.4	O86402, O86404, O86409	
Other viral diseases complicating pregnancy	O98.51	O98.5	O98.51	
Other viral diseases complicating pregnancy, first trimester	O98.511	O98.5	O98.511	
Other viral diseases complicating pregnancy, second trimester	O98.512	O98.5	O98.512	
Other viral diseases complicating pregnancy, third trimester	O98.513	O98.5	O98.513	
Other viral diseases complicating pregnancy, unspecified trimester	O98.519	O98.5	O98.519	
Other viral diseases complicating childbirth	O98.52	O98.5	O98.52	
Other viral diseases complicating the puerperium	O98.53	O98.5	O98.53	
Other maternal infectious and parasitic diseases complicating pregnancy	O98.81	O98.8	O98801-O98804, O98809	
Other maternal infectious and parasitic diseases complicating pregnancy, first trimester	O98.811	O98.8		
Other maternal infectious and parasitic diseases complicating pregnancy, second trimester	O98.812	O98.8		
Other maternal infectious and parasitic diseases complicating pregnancy, third trimester	O98.813	O98.8		
Other maternal infectious and parasitic diseases complicating pregnancy, unspecified trimester	O98.819	O98.8		
Diseases of the respiratory system complicating pregnancy	O99.51	O99.5	O99501-O99504, O99509	
Diseases of the respiratory system complicating pregnancy, first trimester	O99.511	O99.5		
Diseases of the respiratory system complicating pregnancy, second trimester	O99.512	O99.5		

Diseases of the respiratory system complicating pregnancy, third trimester	O99.513	O99.5		
Diseases of the respiratory system complicating pregnancy, unspecified trimester	O99.519	O99.5		
Cough	R05	R05	R05	
Shortness of breath	R06.02	R06.0	R060	
Stridor	R06.1	R06.1	R06.1	
Wheezing	R06.2	R06.2	R06.2	
Tachypnea, not elsewhere classified	R06.82	R06.8	R064	
Chest pain on breathing	R07.1	R07.1	R07.1	
Asphyxia	R09.01	R09.0	R09.01	
Hypoxemia	R09.02	R09.0	R09.02	
Respiratory arrest	R09.2	R09.2	R09.2	
Abnormal sputum	R09.3	R09.3	R09.3	
Other specified symptoms and signs involving the circulatory and respiratory systems	R09.89	R09.89	R09.89	
Fever of other and unknown origin	R50	R50	R50	
Drug induced fever	R50.2	R50.2	R50.2	
Fever presenting with conditions classified elsewhere	R50.81	R50.8	R50.81	
Postprocedural fever	R50.82	R50.8	R50.82	
Postvaccination fever	R50.83	R50.8	R50.83	
Febrile nonhemolytic transfusion reaction	R50.84	R50.8	R50.84	
Fever, unspecified	R50.9	R50.9	R50.9	
Chills (without fever)	R68.83	R68.8 (not specific to chills)	R680	
Pleural effusion, not elsewhere classified	J90	J90		
Acute and chronic respiratory failure	J96.2	No J96.2 – J96.1 (chronic respiratory failure)		

Acute and chronic respiratory failure, unspecified whether with hypoxia or hypercapnia	J96.20	--		
Acute and chronic respiratory failure with hypoxia	J96.21	--		
Acute and chronic respiratory failure with hypercapnia	J96.22	--		
Hemoptysis	R04.2	R04.2		
Dyspnea	R06.0	R06.0		
Dyspnea, unspecified	R06.00	R06.0		
Other forms of dyspnea	R06.09	R06.0		
Nasal congestion	R09.81	Code does not exist		
Severe sepsis with septic shock	R65.21	Code does not exist		
Shock, unspecified	R57.9	R57.9		
Sepsis, unspecified organism	A41.9	A41.9		
Shock during or following labor and delivery	O75.1	O75.1		

Description	ICD-10 Code	ICD-10-AM Code	ICD-10-CA Code	Notes
High Risk Medical Condition Diagnostic Codes				
Blood Disorders				
Iron deficiency anemia secondary to blood loss (chronic)	D50.0	D50.0	D50.0	
Other iron deficiency anemias	D50.8	D50.0	D50.8	
Iron deficiency anemia, unspecified	D50.9	D50.8	D50.9	
Vitamin B12 deficiency anemia	D51	D50.9	D51	
Folate deficiency anemia	D52	D51.0-D51.9	D52	
Other nutritional anemias	D53	D52.0-D52.9	D53	
Other hereditary hemolytic anemias	D58	D53.0-D53.9	D58	
Acquired hemolytic anemia	D59	D58.0-D58.9	D59	
Other aplastic anemias and other bone marrow failure syndromes	D61	D59.0-D59.9	D61	
Anemia in neoplastic disease	D63.0	D61.0-D61.9	D63.0	

Anemia in chronic kidney disease	D63.1	D63	D63	
Anemia in other chronic diseases classified elsewhere	D63.8	D63	D63.8	
Hereditary sideroblastic anemia	D64.0	D63	D64.0	
Secondary sideroblastic anemia due to disease	D64.1	D64.0	D64.1	
Secondary sideroblastic anemia due to drugs and toxins	D64.2	D64.1	D64.2	
Other sideroblastic anemias	D64.3	D64.2	D64.3	
Anemia due to antineoplastic chemotherapy	D64.81	D64.3	D64.81	
Other specified anemias	D64.89	D64.8	D648	
Anemia, unspecified	D64.9	D64.8	D64.9	
Other coagulation defects	D68	D64.9	D68	
Purpura and other hemorrhagic conditions	D69	D68.0-D69.9	D69	
Neutropenia	D70	D69.0-D69.9	D70	
Functional disorders of polymorphonuclear neutrophils	D71	D70	D71	
Other disorders of white blood cells	D72	D71	D72	
Diseases of spleen	D73	D72.0-D72.9	D73	
Methemoglobinemia	D74	D73.0-D73.9	D74	
Other and unspecified diseases of blood and blood-forming organs	D75	D74.0-D74.9	D75	
Other disorders of blood and blood-forming organs in diseases classified elsewhere	D77	D77	D77	
**Diabetes**				
Diabetes mellitus due to underlying condition	E08	Code does not exist	Code does not exist	
Drug or chemical induced diabetes mellitus	E09	E09	Code does not exist	
Type 1 diabetes mellitus	E10	E10	E10	
Type 2 diabetes mellitus	E11	E11	E11	
Other specified diabetes mellitus	E13	E13	E13	
Nondiabetic hypoglycemic coma	E15			
**Heart Disease**				

Candidal endocarditis	B37.6			
Disseminated intravascular coagulation (defibrination syndrome)	D65			
Hypertensive retinopathy	H35.03	H35.0	H35, H350-H359	ICD-10-AM H35.03 does not exist: H35.0 incudes other retinal disorders
Rheumatic fever with heart involvement	I01	I01.0-I01.9	I01	
Rheumatic mitral valve disease	I05	I05.0-I05.9	I05	
Rheumatic aortic valve diseases	I06	I06.0-I06.9	I06	
Other rheumatic heart diseases	I09	I09.0-I09.9	I09	
Hypertensive heart disease	I11	I11.0-I11.9	I11	
Hypertensive chronic kidney disease	I12	I12.0-I12.9	I12	
Hypertensive heart and chronic kidney disease	I13	I13.0-I13.9	I13	
Angina pectoris	I20	I20.0-I20.9	I20	
ST elevation (STEMI) and non-ST elevation (NSTEMI) myocardial infarction	I21	I21.0-I21.9	I21	
Other acute ischemic heart diseases	I24	I24.0-I24.9	I24	
Old myocardial infarction	I25.2	I25.2	I25.2	
Atherosclerosis of coronary artery bypass graft(s) and coronary artery of transplanted heart with angina pectoris	I25.7	Code does not exist	I258	
Pulmonary embolism	I26	I26.0, I26.9	I26	
Other pulmonary heart diseases	I27	I27.0-I27.9	I27	
Other diseases of pulmonary vessels	I28	I28.0-I28.9	I28	
Acute pericarditis	I30	I30.0-I30.9	I30	
Other diseases of pericardium	I31	I31.0-I31.9	I31	
Acute and subacute endocarditis	I33	I33.0, I33.9	I33	
Nonrheumatic mitral valve disorders	I34	I34.0-I34.9	I34	
Nonrheumatic aortic valve disorders	I35	I35.0-I35.9	I35	
Nonrheumatic tricuspid valve disorders	I36	I36.0-I36.9	I36	
Nonrheumatic pulmonary valve disorders	I37	I37.0-I37.9	I37	
Endocarditis, valve unspecified	I38	I38	I38	
Endocarditis and heart valve disorders in diseases classified elsewhere	I39	I39.0-I39.8	I39	

Acute myocarditis	I40	I40.0-I40.9	I40	
Myocarditis in diseases classified elsewhere	I41	I41.0-I41.8	I41	
Cardiomyopathy	I42	I42.0-I42.9	I42	
Cardiac arrest	I46	I46.0-I46.9	I46	
Paroxysmal tachycardia	I47	I47.0-I47.9	I47	
Atrial fibrillation and flutter	I48	I48.0-I48.9	I48	
Other cardiac arrhythmias	I49	I49.0-I49.9	I49	
Heart failure	I50	I50.0-I50.9	I50	
Complications and ill-defined descriptions of heart disease	I51	I51	I51	
Other heart disorders in diseases classified elsewhere	I52	I52	I52	
Atherosclerosis	I70	I70	I70	
Aortic aneurysm and dissection	I71	I71	I71	
Other aneurysm	I72	I72	I72	
Other peripheral vascular diseases	I73	I73	I73	
Arterial embolism and thrombosis	I74	I74	I74	
Atheroembolism	I75	I70.9	Code does not exist	
Other disorders of arteries and arterioles	I77	I77	I77	
Polyarteritis nodosa	M30.0	M30.0	M30.0	
Other necrotizing vasculopathies	M31	M31.0-M31.9	M31	
Congenital malformations of cardiac septa	Q21	Q21.00-Q21.9	Q21	
Congenital malformations of pulmonary and tricuspid valves	Q22	Q22	Q22	
Congenital malformations of aortic and mitral valves	Q23	Q23	Q23	
Other congenital malformations of heart	Q24	Q24	Q24	
Congenital malformations of great arteries	Q25	Q25	Q25	
Congenital malformations of great veins	Q26	Q26	Q26	
Other congenital malformations of peripheral vascular system	Q27	Q27	Q27	
Complications of cardiac and vascular prosthetic devices, implants and grafts	T82	T82	T82	

Presence of cardiac and vascular implants and grafts	Z95	Z95	Z95	
**Cancer**				
Malignant neoplasm of lip	C00	C00	C00	
Malignant neoplasm of base of tongue	C01	C01	C01	
Malignant neoplasm of other and unspecified parts of tongue	C02	C02	C02	
Malignant neoplasm of parotid gland	C07	C07	C07	
Malignant neoplasm of other and unspecified major salivary glands	C08	C08	C08	
Malignant neoplasm of gum	C03	C03	C03	
Malignant neoplasm of floor of mouth	C04	C04	C04	
Malignant neoplasm of palate	C05	C05	C05	
Malignant neoplasm of other and unspecified parts of mouth	C06	C06	C06	
Malignant neoplasm of tonsil	C09	C09	C09	
Malignant neoplasm of oropharynx	C10	C10	C10	
Malignant neoplasm of nasopharynx	C11	C11	C11	
Malignant neoplasm of pyriform sinus	C12	C12	C12	
Malignant neoplasm of hypopharynx	C13	C13	C13	
Malignant neoplasm of other and ill-defined sites in the lip, oral cavity and pharynx	C14	C14	C14	
Malignant neoplasm of esophagus	C15	C15	C15	
Malignant neoplasm of stomach	C16	C16	C16	
Malignant neoplasm of small intestine	C17	C17	C17	
Malignant neoplasm of colon	C18	C18	C18	
Malignant neoplasm of rectosigmoid junction	C19	C19	C19	
Malignant neoplasm of rectum	C20	C20	C20	
Malignant neoplasm of anus and anal canal	C21	C21	C21	
Malignant neoplasm of liver and intrahepatic bile ducts	C22	C22	C22	
Malignant neoplasm of gallbladder	C23	C23	C23	

Malignant neoplasm of other and unspecified parts of biliary tract	C24	C24	C24	
Malignant neoplasm of pancreas	C25	C25	C25	
Malignant neoplasm of retroperitoneum and peritoneum	C48	C48	C48	
Malignant neoplasm of other and ill-defined digestive organs	C26	C26	C26	
Malignant neoplasm of nasal cavity and middle ear	C30	C30	C30	
Malignant neoplasm of accessory sinuses	C31	C31	C31	
Malignant neoplasm of larynx	C32	C32	C32	
Malignant neoplasm of trachea	C33	C33	C33	
Malignant neoplasm of bronchus and lung	C34	C34	C34	
Malignant neoplasm of heart, mediastinum and pleura	C38	C38	C38	
Malignant neoplasm of thymus	C37	C37	C37	
Malignant neoplasm of other and ill-defined sites in the respiratory system and intrathoracic organs	C39	C39	C39	
Malignant neoplasm of bone and articular cartilage of limbs	C40	C40	C40	
Malignant neoplasm of bone and articular cartilage of other and unspecified sites	C41	C41	C41	
Other and unspecified malignant neoplasm of skin	C44	C44	C44	
Malignant neoplasm of other connective and soft tissue	C49	C49	C49	
Malignant Melanoma of Skin	C43	C43	C43	
Kaposi's Sarcoma	C46	C46	C46	
Mesothelioma	C45	C45	C45	
Malignant neoplasm of uterus, part unspecified	C55	C55	C55	
Malignant neoplasm of cervix uteri	C53	C53	C53	
Malignant neoplasm of placenta	C58	C58	C58	
Malignant neoplasm of corpus uteri	C54	C54	C54	

Malignant neoplasm of ovary	C56	C56	C56	
Malignant neoplasm of vulva	C51	C51	C51	
Malignant neoplasm of vagina	C52	C52	C52	
Malignant neoplasm of breast	C50	C50	C50	
Malignant neoplasm of prostate	C61	C61	C61	
Malignant neoplasm of testis	C62	C62	C62	
Malignant neoplasm of penis	C60	C60	C60	
Malignant neoplasm of bladder	C67	C67	C67	
Malignant neoplasm of kidney, except renal pelvis	C64	C64	C64	
Malignant neoplasm of eye and adnexa	C69	C69	C69	
Malignant neoplasm of brain	C71	C71	C71	
Malignant neoplasm of spinal cord, cranial nerves and other parts of central nervous system	C72	C72	C72	
Malignant neoplasm of thyroid gland	C73	C73	C73	
Malignant neoplasm of adrenal gland	C74	C74	C74	
Malignant neoplasm of other endocrine glands and related structures	C75	C75	C75	
Malignant neoplasm of other and ill-defined sites	C76	C76	C76	
Secondary and unspecified malignant neoplasm of lymph nodes	C77	C77	C77	
Secondary malignant neoplasm of respiratory and digestive organs	C78	C78	C78	
Secondary malignant neoplasm of other and unspecified sites	C79	C79	C79	
Malignant neoplasm without specification of site	C80	C80	C80	
Hodgkin lymphoma	C81	C81	C81	
Non-follicular lymphoma	C83	C83	C83	
Multiple myeloma and malignant plasma cell neoplasms	C90	C90	C90	
Lymphoid leukemia	C91	C91	C91	
Myeloid leukemia	C92	C92	C92	

Monocytic leukemia	C93	C93	C93	
Other leukemias of specified cell type	C94	C94	C94	
Leukemia of unspecified cell type	C95	C95	C95	
Malignant neuroendocrine tumors	C7A	Code does not exist	Code does not exist	
Carcinoma in situ of middle ear and respiratory system	D02	D02	D02	
Neoplasm of uncertain behavior of oral cavity and digestive organs	D37	D37	D37	
Neoplasm of uncertain behavior of middle ear and respiratory and intrathoracic organs	D38	D38	D38	
Neoplasm of uncertain behavior of female genital organs	D39	D39	D39	
Neoplasm of uncertain behavior of testis	D40.1	D40.1	D40.1	
Neoplasm of uncertain behavior of prostate	D40.0	D40.0	D40.0	
Neoplasm of uncertain behavior of other specified male genital organs	D40.8	D40.7	D40.7	
Neoplasm of uncertain behavior of male genital organ, unspecified	D40.9	D40.9	D40.9	
Neoplasm of uncertain behavior of kidney	D41.0	D41.0	D41.0	
Neoplasm of uncertain behavior of renal pelvis	D41.1	D41.1	D41.1	
Neoplasm of uncertain behavior of ureter	D41.2	D41.2	D41.2	
Neoplasm of uncertain behavior of urethra	D41.3	D41.3	D41.3	
Neoplasm of uncertain behavior of bladder	D41.4	D41.4	D41.4	
Neoplasm of uncertain behavior of other specified urinary organs	D41.8	D41.7	D41.7	
Neoplasm of uncertain behavior of unspecified urinary organ	D41.9	D41.9	D41.9	
Neoplasm of uncertain behavior of cerebral meninges	D42.0	D42.0	D42.0	
Neoplasm of uncertain behavior of spinal meninges	D42.1	D42.1	D42.1	
Neoplasm of uncertain behavior of meninges, unspecified	D42.9	D42.9	D42.9	

Neoplasm of uncertain behavior of brain, supratentorial	D43.0	D43.0	D43.0	
Neoplasm of uncertain behavior of brain, infratentorial	D43.1	D43.1	D43.1	
Neoplasm of uncertain behavior of brain, unspecified	D43.2	D43.2	D43.2	
Neoplasm of uncertain behavior of cranial nerves	D43.3	D43.3	D43.3	
Neoplasm of uncertain behavior of spinal cord	D43.4	D43.4	D43.4	
Neoplasm of uncertain behavior of other specified parts of central nervous system	D43.8	D43.7	D43.7	
Neoplasm of uncertain behavior of central nervous system, unspecified	D43.9	D43.9	D43.9	
Neoplasm of uncertain behavior of adrenal gland	D44.1	D44.1	D44.1	
Neoplasm of uncertain behavior of thyroid gland	D44.0	D44.0	D44.0	
Neoplasm of uncertain behavior of parathyroid gland	D44.2	D44.2	D44.2	
Neoplasm of uncertain behavior of pituitary gland	D44.3	D44.3	D44.3	
Neoplasm of uncertain behavior of craniopharyngeal duct	D44.4	D44.4	D44.4	
Neoplasm of uncertain behavior of pineal gland	D44.5	D44.5	D44.5	
Neoplasm of uncertain behavior of carotid body	D44.6	D44.6	D44.6	
Neoplasm of uncertain behavior of aortic body and other paraganglia	D44.7	D44.7	D44.7	
Neoplasm of uncertain behavior of unspecified endocrine gland	D44.9	D44.9	D44.9	
Neoplasm of uncertain behavior of breast	D48.6	D48.6	D48.6	
Neoplasm of uncertain behavior of bone and articular cartilage	D48.0	D48.0	D48.0	

Neoplasm of uncertain behavior of connective and other soft tissue	D48.1	D48.1	D48.1	
Neoplasm of uncertain behavior of peripheral nerves and autonomic nervous system	D48.2	D48.2	D48.2	
Neoplasm of uncertain behavior of retroperitoneum	D48.3	D48.3	D48.3	
Neoplasm of uncertain behavior of peritoneum	D48.4	D48.4	D48.4	
Neoplasm of uncertain behavior of skin	D48.5	D48.5	D48.5	
Neoplasm of uncertain behavior of other specified sites	D48.7	D48.7	D48.7	
Neoplasm of uncertain behavior, unspecified	D48.9	D48.9	D48.9	
Encounter for antineoplastic chemotherapy	Z51.11	Z51.11	Z511	
Encounter for antineoplastic immunotherapy	Z51.12	Z51.12	Code does not exist	
Immune				
Human immunodeficiency virus [HIV] disease	B20	B20	B24	
Retrovirus infections, not elsewhere classified	B33.3	B33.3	B33.3	
Pneumocystosis	B59	B59	B59	
Enterobiasis	B80	B80	B80	
Other intestinal helminthiases, not elsewhere classified	B81	B81	B81	
Unspecified intestinal parasitism	B82	B82	B82	
Onchocerciasis without eye disease	B73.1	B73	B73	
Diseases of spleen	D73	D73	D73	
Other specified myoneural disorders	G70.8	G70.8	G70.8	
Systemic lupus erythematosus, organ or system involvement unspecified	M32.10	M32.1	M321	
Other systemic involvement of connective tissue	M35	M35	M35	
Rheumatoid arthritis with rheumatoid factor	M05	M05	M05	

Other rheumatoid arthritis	M06	M06	M06	
Abnormal findings on cytological and histological examination of urine	R82.8	R82.8	R82.8	
Abnormal histological findings in specimens from respiratory organs and thorax	R84.7	R84.7	R84.7	
Abnormal histological findings in specimens from digestive organs and abdominal cavity	R85.7	R85.7	R85.7	
Abnormal histological findings in specimens from male genital organs	R86.7	R86.7	R86.7	
Abnormal histological findings in specimens from female genital organs	R87.7	R87.7	R87.7	
Abnormal histological findings in specimens from other organs, systems and tissues	R89.7	R89.7	R89.7	
Poisoning by, adverse effect of and underdosing of primarily systemic and hematological agents, not elsewhere classified	T45	T45.1	T45	
Asymptomatic human immunodeficiency virus [HIV] infection status	Z21	Z21	Z21	
Transplanted organ and tissue status	Z94	Z94	Z94	
**Renal**				
Anemia in chronic kidney disease	D63.1	Code does not exist	Code does not exist	
Hypertensive chronic kidney disease with stage 5 chronic kidney disease or end stage renal disease	I12.0	I12.0	I12	
Hypertensive heart and chronic kidney disease without heart failure, with stage 5 chronic kidney disease, or end stage renal disease	I13.11	Code does not exist	I13	
Hypertensive heart and chronic kidney disease with heart failure and with stage 5 chronic kidney disease, or end stage renal disease	I13.2	I13.2	Code does not exist	
Hepatorenal syndrome	K76.7			

Nephrotic syndrome	N04	N04	N04	
Chronic nephritic syndrome	N03	N03	N03	
Unspecified nephritic syndrome	N05	N05	N05	
Acute kidney failure	N17	N17	N17	
Chronic kidney disease (CKD)	N18	N18	N18	
Unspecified kidney failure	N19	N19	N19	
Renal sclerosis, unspecified	N26.9	Code does not exist	N26.9	
Renal osteodystrophy	N25.0	N25.0	N25.0	
Nephrogenic diabetes insipidus	N25.1	N25.1	N25.1	
Small kidney, unilateral	N27.0	N27.0	N27.0	
Small kidney, bilateral	N27.1	N27.1	N27.1	
Small kidney, unspecified	N27.9	N27.9	N27.9	
Other specified disorders of kidney and ureter	N28.89	Code does not exist	N2888	
Cystic kidney disease	Q61	Q61	Q61	
Complications of other internal prosthetic devices, implants and grafts	T85	T85	T85	
Encounter for care involving renal dialysis	Z49	Z49	Z49	
Patient's noncompliance with renal dialysis	Z91.15	Code does not exist	Code does not exist	
**Liver**				
Alcoholic liver disease	K70	K70	K70	
Acute and subacute hepatic failure without coma	K72.00			
Hepatic failure, unspecified without coma	K72.90			
Hepatic failure, unspecified with coma	K72.91			
Chronic hepatitis, not elsewhere classified	K73	K73	K73	
Fibrosis and cirrhosis of liver	K74	K74	K74	
Phlebitis of portal vein	K75.1	K75.1	K75.1	
Nonspecific reactive hepatitis	K75.2	K75,2	K75.2	
Granulomatous hepatitis, not elsewhere classified	K75.3	K75.3	K75.3	
Autoimmune hepatitis	K75.4	K75.4	K75.4	
Other specified inflammatory liver diseases	K75.8	K75.8	K75.8	
Inflammatory liver disease, unspecified	K75.9	K75.9	K75.9	

Central hemorrhagic necrosis of liver	K76.2			
Infarction of liver	K76.3			
**Asthma**				
Chronic obstructive pulmonary disease with acute lower respiratory infection	J44.0			
Chronic obstructive pulmonary disease with acute exacerbation	J44.1			
Asthma	J45	J45	J45	
**Other Lung**				
Respiratory tuberculosis	A15	A15	A15	
Tuberculosis of nervous system	A17	A17	A17	
Tuberculosis of other organs	A18	A18	A18	
Miliary tuberculosis	A19	A19	A19	
Infection due to other mycobacteria	A31	A31	A31	
Sarcoidosis	D86	D86	D86	
Cystic fibrosis	E84	E84	E84	
Bronchitis, not specified as acute or chronic	J40	J40	J40	
Simple and mucopurulent chronic bronchitis	J41	J41	J41	
Unspecified chronic bronchitis	J42	J42	J42	
Emphysema	J43	J43	J43	
Bronchiectasis	J47	J47	J47	
Coalworker's pneumoconiosis	J60	J60	J60	
Pneumoconiosis due to asbestos and other mineral fibers	J61	J61	J61	
Pneumoconiosis due to talc dust	J62.0	J62.0	J62.0	
Pneumoconiosis due to other dust containing silica	J62.8	J62.8	J62.8	
Pneumoconiosis due to other specified inorganic dusts	J63.6	J63.8	J638	
Unspecified pneumoconiosis	J64	J64	J64	
Hypersensitivity pneumonitis due to organic dust	J67	J67	J67	
Respiratory conditions due to inhalation of chemicals, gases, fumes and vapors	J68	J68	J68	

Pneumonitis due to inhalation of food and vomit	J69.0	J69.0	J69.0	
Pneumonitis due to inhalation of oils and essences	J69.1	J69.1	J69.1	
Pneumonitis due to inhalation of other solids and liquids	J69.8	J69.8	J69.8	
Respiratory conditions due to unspecified external agent	J70.9	J70.9	J70.9	
Abscess of lung and mediastinum	J85	J85	J85	
Pneumothorax and air leak	J93	J93	J93	
Other respiratory disorders	J98	J98	J98	
Systemic sclerosis [scleroderma]	M34	M34	M34	
Other systemic involvement of connective tissue	M35	M35	M35	
Other congenital malformations of respiratory system	Q34	Q34	Q34	
Pleurisy	R09.1	R09.1	R09.1	
Obesity				
Overweight and obesity	E66	E66	E66	
Obesity complicating pregnancy, unspecified trimester	O99.210	Code does not exist	Code does not exist	
Obesity complicating pregnancy, first trimester	O99.211	Code does not exist	Code does not exist	
Obesity complicating pregnancy, second trimester	O99.212	Code does not exist	Code does not exist	
Obesity complicating pregnancy, third trimester	O99.213	Code does not exist	Code does not exist	
Obesity complicating childbirth	O99.214	Code does not exist	Code does not exist	
Obesity complicating the puerperium	O99.215	Code does not exist	Code does not exist	
Bariatric surgery status complicating pregnancy, unspecified trimester	O99.840	Code does not exist	Code does not exist	
Bariatric surgery status complicating pregnancy, first trimester	O99.841	Code does not exist	Code does not exist	
Bariatric surgery status complicating pregnancy, second trimester	O99.842	Code does not exist	Code does not exist	
Bariatric surgery status complicating pregnancy, third trimester	O99.843	Code does not exist	Code does not exist	

Bariatric surgery status complicating childbirth	O99.844	Code does not exist	Code does not exist	
Bariatric surgery status complicating the puerperium	O99.845	Code does not exist	Code does not exist	
**Other Metabolic**				
Other hypothyroidism	E03	E03	E03	
Other nontoxic goiter	E04	E04	E04	
Thyrotoxicosis [hyperthyroidism]	E05	E05	E05	
Thyroiditis	E06	E06	E06	
Other disorders of thyroid	E07	E07	E07	
Other disorders of pancreatic internal secretion	E16	E16	E16	
Hyperfunction of pituitary gland	E22	E22	E22	
Hypofunction and other disorders of the pituitary gland	E23	E23	E23	
Diseases of thymus	E32	E32	E32	
Other disorders of adrenal gland	E27	E27	E27	
Kwashiorkor	E40	E40	E40	
Nutritional marasmus	E41	E41	E41	
Marasmic kwashiorkor	E42	E42	E42	
Unspecified severe protein-calorie malnutrition	E43	E43	E43	
Protein-calorie malnutrition of moderate and mild degree	E44	E44	E44	
Retarded development following protein-calorie malnutrition	E45	E45	E45	
Unspecified protein-calorie malnutrition	E46	E46	E46	
Vitamin A deficiency	E50	E50	E50	
Thiamine deficiency	E51	E51	E51	
Niacin deficiency [pellagra]	E52	E52	E52	
Deficiency of other B group vitamins	E53	E53	E53	
Ascorbic acid deficiency	E54	E54	E54	
Vitamin D deficiency	E55	E55	E55	
Other vitamin deficiencies	E56	E56	E56	

Disorders of aromatic amino-acid metabolism	E70	E70	E70	
Disorders of branched-chain amino-acid metabolism and fatty-acid metabolism	E71	E71	E71	
Other disorders of amino-acid metabolism	E72	E72	E72	
Other disorders of carbohydrate metabolism	E74	E74	E74	
Disorders of lipoprotein metabolism and other lipidemias	E78	E78	E78	
Other and unspecified metabolic disorders	E88	E88	E88	
Gout	M10	M10	M10	
Disorders of mineral metabolism	E83	E83	E83	
**Neurologic**				
Tuberculosis of nervous system	A17	A17	A17	
Atypical virus infections of central nervous system	A81	A81	A81	
Sequelae of central nervous system tuberculosis	B90.0	B90.0	B90.0	
Vascular dementia	F01	F01	F01	
Dementia in other diseases classified elsewhere	F02	F02	F02	
Unspecified dementia	F03	F03	F03	
Personality and behavioral disorders due to known physiological condition	F07	F07	F07	
Moderate intellectual disabilities	F71	F71	F71	
Severe intellectual disabilities	F72	F72	F72	
Profound intellectual disabilities	F73	F73	F73	
Disorders of sphingolipid metabolism and other lipid storage disorders	E75	E75	E75	
Parkinson's disease	G20	G20	G20	
Other extrapyramidal and movement disorders	G25	G25	G25	
Extrapyramidal and movement disorders in diseases classified elsewhere	G26	G26	G26	
Hereditary ataxia	G11	G11	G11	
Spondylosis	M47	M47	M47	
Hereditary and idiopathic neuropathy	G60	G60	G60	

Multiple sclerosis	G35	G35	G35	
Other acute disseminated demyelination	G36	G36	G36	
Inflammatory polyneuropathy	G61	G61	G61	
Other and unspecified polyneuropathies	G62	G62	G62	
Hemiplegia and hemiparesis	G81	G81	G81	
Cerebral palsy	G80	G80	G80	
Paraplegia (paraparesis) and quadriplegia (quadriparesis)	G82	G82	G82	
Epilepsy and recurrent seizures	G40	G40	G40	
Other disorders of brain	G93	G93	G93	
Other disorders of central nervous system	G96	G96	G96	
Rheumatic chorea	I02	I02	I02	
Nontraumatic subarachnoid hemorrhage	I60	I60	I60	
Nontraumatic intracerebral hemorrhage	I61	I61	I61	
Other and unspecified nontraumatic intracranial hemorrhage	I62	I62	I62	
Occlusion and stenosis of precerebral arteries, not resulting in cerebral infarction	I65	I65	I65	
Occlusion and stenosis of cerebral arteries, not resulting in cerebral infarction	I66	I66	I66	
Cerebral ischemia	I67.82	Code does not exist	G45	
Cerebrovascular disease, unspecified	I67.9	I67.9	I67.9	
Sequelae of cerebrovascular disease	I69	I69	I69	
Myotonic chondrodystrophy	G71.13	G71.1	Code does not exist	
Convulsions, not elsewhere classified	R56	R56	R56	
Complications of other internal prosthetic devices, implants and grafts	T85	T85	T85	
Transient global amnesia	G45.4			
Somnolence	R40.0			
Transient alteration of awareness	R40.4			
Altered mental status, unspecified	R41.82			
Auditory hallucinations	R44.0			
Hypertensive encephalopathy	I67.4			
Salmonella meningitis	A02.21			
Meningococcal infection	A39			

Enteroviral meningitis	A87.0			
Adenoviral meningitis	A87.1			
Candidal meningitis	B37.5			
Coccidioidomycosis meningitis	B38.4			
Bacterial meningitis, not elsewhere classified	G00			
Meningitis in bacterial diseases classified elsewhere	G01			
Meningitis in other infectious and parasitic diseases classified elsewhere	G02			
Meningitis due to other and unspecified causes	G03			
Encephalitis, myelitis, and encephalomyelitis in diseases classified elsewhere	G05			
Acute transverse myelitis in demyelinating disease of central nervous system	G37.3			
Unspecified coma	R40.20			
Persistent vegetative state	R40.3			
Persistent migraine aura with cerebral infarction	G43.6			
Cerebral infarction	I63			
Septic arterial embolism	I76			
Muscular				
Myopathy	G72.81			
Myositis	M60			
Rhabdomyolysis	M62.82			

Description	ICD-10 Code	ICD-10-AM	ICD-10-CM	Notes
Pregnancy Complication Diagnostic Codes				
Chlamydial lymphogranuloma (venereum)	A55	A55	A55	
Anogenital herpesviral (herpes simplex) infections	A60	A60	A60	

Chlamydia psittaci infections	A70	A70	A70	
Rabies	A82	A82	A82	
Mosquito-borne viral encephalitis	A83	A83	A83	
Tick-borne viral encephalitis	A84	A84	A84	
Other viral encephalitis, not elsewhere classified	A85	A85	A85	
Unspecified viral encephalitis	A86	A86	A86	
Dengue fever (classical dengue)	A90	A90	A90	
Dengue hemorrhagic fever	A91	A91	A91	
Other mosquito-borne viral fevers	A92	A92	A92	
Yellow fever	A95	A95	A95	
Arenaviral hemorrhagic fever	A96	A96	A96	
Other viral hemorrhagic fevers, not elsewhere classified	A98	A98	A98	
Varicella meningitis	B01.0	B01.0	B01.0	
Varicella encephalitis and encephalomyelitis	B01.11	B01.1	B011	
Varicella myelitis	B01.12	B01.1	Code does not exist	
Varicella pneumonia	B01.2	B01.2	B01.2	
Varicella keratits	B01.81	B01.8	B018, B019	
Other varicella complications	B01.89	B01.8	Code does not exist	
Other herpes zoster eye disease	B02.39	B02.3	B023	
Acute hepatitis A	B15	B15	B15	
Acute hepatitis B	B16	B16	B16	
Other acute viral hepatitis	B17	B17	B17	
Chronic viral hepatitis	B18	B18	B18	
Unspecified viral hepatitis	B19	B19	B19	
Cytomegaloviral disease	B25	B25	B25	
Infectious mononucleosis	B27	B27	B27	
Coxsackievirus as the cause of disease classified elsewhere	B97.11	Code does not exist	B97.11	
Hemoglobinuria due to hemolysis from other external causes	D59.6	D59.6	D59.6	

Amnestic disorder due to known physiological condition	F04	F04.00-F04.9	F04	
Delirium due to known physiological condition	F05	F05	F05	
Other mental disorders due to known physiological condition	F06	F06	F06	
Alcohol related disorders	F10	F10	F10	
Opioid related disorders	F11	F11	F11	
Cannabis related disorders	F12	F12	F12	
Sedative, hypnotic, or anxiolytic related disorders	F13	F13	F13	
Cocaine related disorders	F14	F14	F14	
Other stimulant related disorders	F15	F15	F15	
Hallucinogen related disorders	F16	F16	F16	
Inhalant related disorders	F18	F18	F18	
Other psychoactive substance related disorders	F19	F19	F19	
Schizophrenia	F20	F20	F20	
Unspecified psychosis not due to a substance or known physiological condition	F29	F29	F29	
Manic episode	F30	F30	F30	
Bipolar disorder	F31	F31	F31	
Major depressive disorder, single episode	F32	F32	F32	
Major depressive disorder, recurrent	F33	F33	F33	
Persistent mood (affective) disorders	F34	F34	F34	
Reaction to severe stress, and adjustment disorders	F43	F43	F43	
Other specified nonpsychotic mental disorders	F48.8	F48.8	F48.8	
Nonpsychotic mental disorder, unspecified	F48.9	F48.9	F48.9	
Puerperal psychosis	F53	F53	F53	
Specific personality disorders	F60	F60	F60	
Other specified disorders of adult personality and behavior	F68.8	F68.8	F68.8	

Unspecified disorder of adult personality and behavior	F69	F69	F69	
Autistic disorder	F84.0	F84.0	F84.0	
Other childhood disintegrative disorder	F84.3	F84.3	F84.3	
Other pervasive developmental disorders	F84.8	F84.8	F84.8	
Pervasive developmental disorder, unspecified	F84.9	F84.9	F84.9	
Portal vein thrombosis	I81	I81	I81	
Other venous embolism and thrombosis	I82	I82	I82	
Other disorders of veins	I87	I87	I87	
Hypotension	I95	I95	I95	
Postinfective and reactive arthropathies	M02	M02	M02	
Recurrent pregnancy loss	N96	N96	N96	
Pre-existing hypertension complicating pregnancy, childbirth and the puerperium	O10	O10.0-O10.9	O10	
Pre-existing hypertension with pre-eclampsia	O11	O11	O11	
Gestational (pregnancy-induced) edema and proteinuria without hypertension	O12	O12	O12	
Gestational (pregnancy-induced) hypertension without significant proteinuria	O13	O13	O13	
Pre-eclampsia	O14	O14	O14	
Eclampsia	O15	O15	O15	
Unspecified maternal hypertension	O16	O16	O16	
Threatened abortion	O20.0	O20.0	O20.0	
Other hemorrhage in early pregnancy	O20.8	O20.8	O20.8	
Hemorrhage in early pregnancy, unspecified	O20.9	O20.9	O20.9	
Excessive vomiting in pregnancy	O21	O21	O21	
Diabetes mellitus in pregnancy, childbirth, and the puerperium	O24	O24	O24	
Maternal care for other conditions predominantly related to pregnancy	O26	O26	O26201, O26202, O26203	
Multiple gestation	O30	O30	O30	
Complications specific to multiple gestation	O31	O31	O31	

Maternal care for malpresentation of fetus	O32	O32	O32	
Maternal care for disproportion	O33	O33	O33	
Maternal care for abnormality of pelvic organs	O34	O34	O34	
Maternal care for (suspected) damage to fetus by drugs	O35.5	O35.5	O35501, O35503, O35509	
Maternal care for known or suspected poor fetal growth	O36.5	O36.5	O36.5	
Polyhydramnios	O40	O40	O40	
Other disorders of amniotic fluid and membranes	O41	O41	O41	
Premature rupture of membranes	O42	O42	O42	
Placental disorders	O43	O43	O43	
Placenta previa	O44	O44	O44101, O44103, O44109	
Premature separation of placenta (abruptio placentae)	O45	O45	O45	
Prolonged pregnancy	O48.1	O48	O48	
Preterm labor	O60	O60	O60	
Failed induction of labor	O61	O61	O61	
Abnormalities of forces of labor	O62	O62	O62	
Long labor	O63	O63	O63	
Obstructed labor due to malposition and malpresentation of fetus	O64	O64	O64	
Obstructed labor due to maternal pelvic abnormality	O65	O65	O65	
Other obstructed labor	O66	O66	O66	
Labor and delivery complicated by intrapartum hemorrhage, not elsewhere classified	O67	O67	O67	
Labor and delivery complicated by abnormality of fetal acid-base balance	O68	O68	O68	
Labor and delivery complicated by umbilical cord complications	O69	O69	O69	
Puerperal sepsis	O85	O85	O85	

Description	ICD-10 Code	ICD-10-AM	ICD-10-CM	Notes
Postpartum thyroiditis	O90.5	O90.5	O90.5	
Tuberculosis complicating pregnancy	O98.01	O98.0	O98001-O98004, O98009	
Tuberculosis complicating childbirth	O98.02	O98.0	Code does not exist	
Tuberculosis complicating the puerperium	O98.03	O98.0	O98001-O98004, O98009	
Syphilis complicating pregnancy	O98.11	O98.1	O98101, O98102, O98103, O98104, O98109	
Gonorrhea complicating pregnancy	O98.21	O98.2	O98201, O98202, O98203, O98204, O98209	
Gonorrhea complicating childbirth	O98.22	O98.2	O98201, O98202, O98203, O98204, O98209	
Gonorrhea complicating the puerperium	O98.23	O98.2	O98201, O98202, O98203, O98204, O98209	
Human immunodeficiency virus (HIV) disease complicating pregnancy	O98.71	O98.7	O98701, O98702, O98703, O98704, O98709	
Drug use complicating pregnancy, childbirth, and the puerperium	O99.32	Code does not exist	Code does not exist	
Tobacco use disorder complicating pregnancy, childbirth, and the puerperium	O99.33	Code does not exist	O99.33	
Diseases of the nervous system complicating pregnancy, childbirth, and the puerperium	O99.35	O99.3	O99.35	
Diseases of the circulatory system complicating pregnancy, childbirth, and the puerperium	O99.4	O99.4	O99401-O99404, O99409	
Pleurodynia	R07.81	Code does not exist	R07.81	
Hemoglobinuria	R82.3	R82.3	Code does not exist	

Hospital Complication Diagnostic Codes				
Asthma Exacerbation	J44.0, J44.1, J45.21, J45.22, J45.901, J45.902	J44.0, J44.1		
Acute renal failure	I13.11, I13.2, K76.7, N17	I13.2, K76.7, N17.0-N17.9		
Liver failure	K72.00, K76.3, K76.2	K76.3, K76.2		
Heart failure	I50.21, I50.23, I50.31, I50.33, I50.41, I50.43	Codes do not exist		
Arterial or venous embolism	I74, I76, I82	I74, I82		
Carditis	A39.50, B37.6, I01.0, I01.1, I01.2, I30, I33, I40, I51.4	B37.6, I01.0, I01.1, I01.2, I30, I33, I40, I51.4		
Cardiac Arrest	I46.9	I46.9		
Altered mental state	F05, F06.0, F06.1, F06.2, F06.30, F06.4, F06.8, G45.4, R40.0, R40.4, R41.82, R44.0	F05, F06.0, F06.1, F06.2, F06.30, F06.4, F06.8, G45.4, R40.0, R44.0		
Encephalitis	G93.4, I67.4, K72.90, K72.91	G93.4, I67.4		
Meningitis	A02.21, A39, A87.0, A87.1, B37.5, B38.4, G00, G01, G02, G03	A39, A87.0, A87.1, B37.5, B38.4, G00, G01, G02, G03		
Anoxic brain damage	G93.1	G93.1		
Encephalitis or myelitis	A39.81, G05, G37.3	G05, G37.3		
Coma	R40.20, R40.3	R40.2		
Status epilepticus	G40.A01, G40.A09, G40.A11, G40.A19, G40.301	Codes do not exist		
Polyneuropathy	G62.81	Code does not exist		ICD-10-AM G63.0 is polyneuropathy with infectious origin
Rhabdomyolsis	M62.82	M62.82		
Myositis	M60	M60		
Myopathy	G72.81	Code does not exist		
Cerebral infarction	G43.6, I63.019, I63.119, I63.139, I63.20, I63.219, I63.22, I63.239, I63.30, I63.40, I63.50, I63.59	Codes do not exist		ICD-10-AM I63 is cerebral infarction – but not as specific as these ICD-10 codes
Cerebral hemorrhage	I60.9, I61.9, I62	I60.9, I61.9, I62		

Cerebral edema	G93.6	G93.6		
Ketaoacidosis	E08.1, E10.1, E13.1	E10.11, E10.12, E10.15, E10.16, E11.11, E11.12, E11.15, E11.16, E13.11, E13.12, E13.15, E13.16, E14.11, E13.12, E14.15, E14.16		
Hyperosmolarity	E08.0, E11.0, E13.0	E11.01, E11.02, E13.01, E13.02, E14.01, E14.02		
Diabetic coma	E08.11, E08.641, E09.11, E09.641, E10.11, E11.01, E11.641, E13.11, E13.641, E15	E10.12, E10.14, E10.16, E11.02, E11.12, E11.14, E11.16, E13.02, E13.12, E13.14, E13.16, E14.02, E14.12, E14.14, E14.16, E15		
Thyroid storm	E05.01, E05.11, E05.21, E05.31, E05.41, E05.81, E05.91	E05.5		
Defibrination syndrome	D65	D65		

i
